# The LAM of the Rings: Large Amplitude Motions in Aromatic Molecules Studied by Microwave Spectroscopy

**DOI:** 10.3390/molecules27123948

**Published:** 2022-06-20

**Authors:** Ha Vinh Lam Nguyen, Walther Caminati, Jens-Uwe Grabow

**Affiliations:** 1 Univ Paris Est Creteil and Université Paris Cité, CNRS, LISA, F-94010 Créteil, France; 2 Institut Universitaire de France (IUF), F-75231 Paris, France; 3 Departimento di Chimica ‘G. Ciamican’, Universita degli Studi di Bologna, Via Selmi 2, 40126 Bologna, Italy; walther.caminati@unibo.it; 4 Institut für Physikalische Chemie und Elektrochemie, Gottfried-Wilhelm-Leibniz-Universität Hannover, Callinstraße 3A, 30167 Hannover, Germany

**Keywords:** rotational spectroscopy, aromatic rings, internal rotation, inversion tunneling, coupled large amplitude motions

## Abstract

Large amplitude motions (LAMs) form a fundamental phenomenon that demands the development of specific theoretical and Hamiltonian models. In recent years, along with the strong progress in instrumental techniques on high-resolution microwave spectroscopy and computational capacity in quantum chemistry, studies on LAMs have become very diverse. Larger and more complex molecular systems have been taken under investigation, ranging from series of heteroaromatic molecules from five- and six-membered rings to polycyclic-aromatic-hydrocarbon derivatives. Such systems are ideally suited to create families of molecules in which the positions and the number of LAMs can be varied, while the heteroatoms often provide a sufficient dipole moment to the systems to warrant the observation of their rotational spectra. This review will summarize three types of LAMs: internal rotation, inversion tunneling, and ring puckering, which are frequently observed in aromatic five-membered rings such as furan, thiophene, pyrrole, thiazole, and oxazole derivatives, in aromatic six-membered rings such as benzene, pyridine, and pyrimidine derivatives, and larger combined rings such as naphthalene, indole, and indan derivatives. For each molecular class, we will present the representatives and summarize the recent insights on the molecular structure and internal dynamics and how they help to advance the field of quantum mechanics.

## 1. Introduction

Microwave spectroscopy has an almost one-century history in which large amplitude motion (LAM) has been a classic topic from the beginning and has especially flourished in recent decades [1,2,3,4,5,6,7]. The first microwave spectrum was that of ammonia recorded by Cleeton and Williams in 1934, showing evidence of an inversion tunneling of the nitrogen atom through the plane spanned by the three hydrogens [8]. In the early years, the microwave spectra of many small organic compounds such as methanol already revealed splittings due to methyl internal rotation [9], or a combination of methyl internal rotation and inversion tunneling as in methyl amine [10]. LAM is also commonly reported in the form of ring puckering [2] with tetrahydrofuran [11] as the classic example. Among a large variety of LAMs, the internal rotation of methyl groups is probably the most extensively investigated, since the cases are numerous. Many studies on the inversion-tunneling motion of a molecular fragment against the rest of the molecule are also reported. The microwave spectra of molecules featuring such a LAM include fine tunneling structures and are significantly more complicated than those of rigid-rotor molecules.

**Figure 1:**
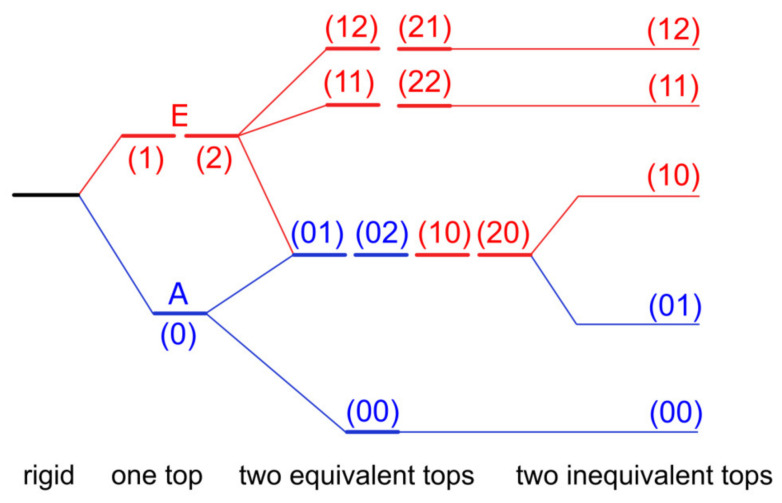
Schematic energy-level diagram illustrating the splittings caused by internal rotation for (i) a rigid rotor (no torsional splitting), (ii) a molecule containing one methyl rotor (splitting into an A and a doubly degenerate E torsional-symmetry species), (iii) a molecule containing two equivalent methyl rotors (splitting into four torsional species), and (iv) a molecule containing two inequivalent methyl rotors (splitting into five torsional species). The three torsional states of the methyl top are given as σ = 0, 1, 2 [28]. The (11) and (12) species are not degenerate due to top–top interactions.

If one methyl internal rotor is involved, energy levels of each rotational state are separated into two, i.e., A and E symmetry species, as depicted in Figure 1 for the vibrational ground state v = 0. In the microwave spectrum, the observed splittings, called A–E splittings, depend on the orientation of the methyl rotor in the molecule, but also on the potential barrier hindering its torsion [12]. On the one hand, if the barrier height is infinite, we can describe the methyl internal rotation as three harmonic oscillators. Their rotational levels are degenerate and no A–E splittings exist in the spectrum. The molecules are semi-rigid rotors: Their microwave spectra can be modeled with sufficient accuracy using a rigid-rotor Hamiltonian model supplemented by centrifugal distortion corrections, as shown by a myriad of previous high-resolution rotational-spectroscopic studies in the literature. On the other hand, if the barrier vanishes, we approach the limit of free internal rotation; for example, the methyl group in CH_3_–C≡C–CF_3_ [13]. For such a molecule, all but the first energy levels are two-fold degenerate. For many intermediate cases between the free internal rotor and the harmonic oscillator, resolvable A–E splittings in the microwave spectra have been reported, and the focus is often on the molecular structures and internal dynamics. Among them, the most extensively studied molecules are those with one methyl rotor, i.e., methacrylic acid [14], methyl vinyl ketone [15], 2-butynoic acid [16], *N*-methylformamide [17], methyl cyanoacetate [18], methyl valerate [19], and methyl hexanoate [20]. The number of studies significantly decreases if multiple methyl internal rotors are present in the molecule. While for two-top cases we can still find some dozens of investigations, for example 3-penten-2-one [21], *N*-acetyl alanine methyl ester [22], and *N*-methylacetamide [23], only a handful of molecules featuring three methyl rotors were reported in the literature [24,25,26]. We found only one four-top molecule, 2,3,4,5-tetramethylthiophene, in an on-going study [3,27]. Multiple methyl internal rotors cause complicated spectral patterns with torsional splittings depending on the number of methyl groups and their interactions. For example, the fine structures of a two-top molecule consist of quartets if the methyl tops are equivalent, as illustrated in Figure 1 where the three torsional states of the methyl top are given as σ = 0, 1, 2. If they are not, quintets are observed, since the (01), (02) and (10), (20) levels are no longer degenerate [28].

**Figure 2:**
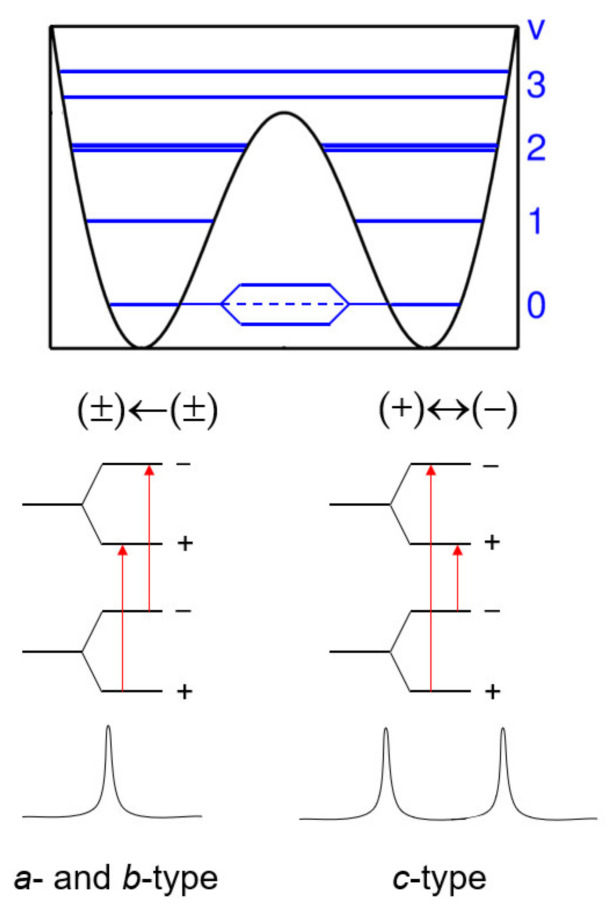
Schematic energy-level diagram illustrating the splittings caused by an inversion-tunneling motion where the *μ_c_* dipole-moment component changes its sign upon tunneling.

If an inversion-tunneling motion is involved in a double minimum energy potential, then the energy levels of a molecule split into (+) and (−) parity states due to a symmetric and an *anti*-symmetric wave function, separated by ∆*E* (see Figure 2) [12]. Ammonia, NH_3_, is the classic example to demonstrate inversion tunneling. It concerns the “umbrella” motion of the nitrogen atom through a plane spanned by three hydrogen atoms [8] connecting two energetically equivalent configurations. These two forms are separated by a potential barrier with a height on the order of 2023 cm^−1^, causing splittings of all rotational transitions of a certain symmetry [29]. For an asymmetric top, the selection rule is that (+)←(−) and (−)←(+) inter-state *g*-type transitions (*g* = *a*, *b*, *c*) occur if the direction of the *μ_g_* dipole-moment component inverts upon inversion. Otherwise, the intra-state selection rule (±)←(±) applies. At zeroth order, those transitions do not split. However, small splittings, referred to as v_t_ = 0 and v_t_ = 1, might be found due to Coriolis interactions, if the spectral resolution allows for their observation.

Through the long history of microwave spectroscopy, considerably fewer studies on inversion tunneling have been reported compared to the large number of investigations of methyl internal rotation. While the symmetry of the frame can be C_1_ for internal rotation, it must be C_s_ or higher to enable a double minimum potential. Furthermore, even if allowed by the molecular symmetry, inversion tunneling is not always isolated, but often coupled with internal rotation. The inversion of the two hydrogen atoms in primary amines is a typical example, which is always accompanied by the internal rotation of the entire amino –NH_2_ group [30,31,32,33]. However, though not occurring as frequently as internal rotation, many molecules of great interest and importance feature this tunneling effect, such as hydrazine [34], ethylene diamine [35], *gauche*-1,3-butadiene [36], phenol [37], acetocyanhydrin [38], and especially dimers in particular [39,40] and complexes in general [41,42,43,44], making inversion tunneling an important LAM subject in rotational spectroscopy.

The potential energy surfaces of ring-puckering motions observed for saturated four- and five-membered rings are quite similar to that of an inversion-tunneling motion in the sense that a double minimum potential is often involved due to the presence of two equivalent conformations [2]. The vibrational interactions cause a slight separation between energy levels, and doublets are observed in the spectrum. In most cases, the splittings are more manageable than those arising from inversion tunneling. Two prototype molecules are cyclobutane [45,46], representative of the four-member rings, and cyclopentene [47], representative of the five-member rings, where the barriers hindering the ring-puckering motion have been determined to be 505 cm^−1^ and 242 cm^−1^, respectively. The number of studies on this type of LAM is not as exhaustive as that of methyl internal rotation, but rather comparable to that of inversion tunneling.

The rotational spectra of many molecules undergoing LAMs were studied in the micron region [48], the millimeter-wave and THz ranges throughout the infrared ranges [49,50,51,52,53], and up to visible/UV [54]. Especially, far-infrared spectroscopy has significantly contributed to the understanding of LAMs [55,56,57,58,59,60,61]. The barriers to methyl internal rotation, for example, are typically between 0 cm^−1^ (free internal rotation) and 1000 cm^−1^, and therefore fall in the far-infrared range. Using this technique, fundamental torsional transitions between the internal rotational energy levels, if active, can be observed directly. Nevertheless, microwave spectroscopy covers by far the most suitable frequency domain to pursue the initial observation and assignment of the internal motions of a molecule in the gas phase. It is an analytical method that has been well-established to extract information on conformational structures of small to medium-sized organic molecules since rotational transitions with low *J* and *K*, which are easier to assign, often fall in this frequency range. The microwave spectra are conformationally specific and enable the characterization of individual conformers [12]. Studying the structures and internal dynamics of a molecule that are relevant to topics as diverse as astrophysics, molecular biology, and environmental sciences using microwave spectroscopy has a long tradition and is still a research field with great potential yet to be exploited. The combination of microwave spectroscopy and theoretical studies has become particularly successful in the last two decades in decoding the spectra of molecules with LAMs and has especially provided reference data for astrophysical research [62,63,64], atmospheric chemistry [65,66,67], and general applications in physical chemistry [68,69,70]. Regarding the resolution of the spectroscopic instruments, during the seventies, a typical high-resolution microwave spectrometer was to use the Stark modulated absorption technique on static gases with a resolution of about 250 kHz (corresponding to a measurement accuracy of 25 kHz) [71]. The resolution has been significantly improved using the impulse excitation technique on pulsed-jet expansions [72]. The electric dipole interaction of the molecular sample occurs while exposure to a standing wave field of the microwave radiation propagating in a “transverse electric magnetic (TEM)”-mode of a Fabry–Pérot-type resonator. Its geometric and electric parameters have a decisive impact on the sensitivity of the spectrometer. A confocal Fabry–Pérot-type resonator is formed by two spherical mirrors of equal curvature at distance *d*, typically made of aluminum. A mirror hosts the nozzle, which, in its simplest form, consists of a circular orifice of 0.5–2.0 mm diameter with an exit channel of 2 mm length conically widening to 4 mm [73]. Applying the coaxial arrangement between the resonator and the molecular beam (COBRA-type) [74], the experimental accuracy currently achievable is 2 kHz [75]. With this extremely high resolution, very small splittings can be resolved.

The time requirements for recording survey spectra have been drastically reduced in the last decade using the chirped (CP) excitation method, which relies on a very short but powerful frequency-ramp signal with a band width of 1 GHz or even more [76]. If the frequency of an electromagnetic field is swept through a molecular resonance in a short time compared to the relaxation time, the so-called fast passage excitation occurs. Even though the molecules are in resonance only for a very short time, a surprisingly large change in the population difference of the states in resonance and in the coherence of the two-level ensemble can be achieved, resulting in a detectable oscillating macroscopic polarization. With all the experimental advances in both resolution and survey speed combined with the rapidly growing computational capacity in the last decades, microwave spectroscopy is extending its key role in yielding precise information on various physical and chemical objectives with its capability to observe the spectra of heavier and larger molecules and even finer splittings arising from quantum-mechanical tunneling effects.

Among the molecules displaying LAM(s), those with conjugated *π*-double bonds are particularly interesting because *π*-electrons can electronically transfer structural and dynamic information across a longer range. This often enables interactions between the LAMs and other fragments of the molecules. Selected examples include studies on a number of benzene derivatives featuring two methyl internal rotations such as 4-methylacetophenone [77], three isomers of dimethylfluorobenzene [78,79,80], three isomers of dimethylbenzaldehyde [81], three isomers of dimethylanisole [82,83,84], or some five-membered aromatic rings such as 4,5-dimethylthiazole [85], 2-acetyl-5-methylfuran [86], and the 2,5-isomers of dimethylpyrrole [87], dimethylfuran [88], and dimethylthiophene [89]. In these studies, it was proven that steric hindrance arising from a neighboring substituent increases the torsional barrier of a methyl group attached to the aromatic ring, but also that related effects can be electronically transferred across the *π*-conjugated system and influence the methyl torsion. While it is easy to predict whether steric effects will occur, the various natures of electronic interactions have neither been exactly understood nor fully explored yet, mainly due to the very limited number of studies on aromatic heterocycles containing LAMs. Notably, hindered methyl internal rotation in aromatic molecules is also a subject of interest for many far-infrared spectroscopic studies, both in the gas phase such as those on *N*-methylaziridine [90], anisole derivatives [91], benzaldehyde, benzoyl fluoride, benzoyl chloride and acetophenone [92] or in the condensed phase [93]. Though less accurate in barrier determination than microwave spectroscopy, the direct information obtained from the transitions between different torsional states together with the parameters on molecular structures also yield accurately determined potential functions.

Unlike the two recent reviews of microwave spectroscopy, one with a focus on internal rotation [94] and one on inversion tunneling [4], this review aims to provide a summary of molecules in which aromatic rings are involved in combination with internal rotations of one or several methyl rotors or inversion-tunneling and ring-puckering motions for a better overview and a better understanding of the long-range electronic effects. For internal rotation, such aromatic rings are ideal to host methyl rotors with sufficiently low barriers hindering internal rotation, such that torsional fine splittings are resolvable. For inversion-tunneling motions, the planarity of an aromatic ring fulfils the symmetry condition required to obtain a double minimum potential and increases the possibility that this rarely explored, but not uncommon quantum-mechanical tunneling effect occurs. The same reason applies to ring-puckering motions where two equivalent configurations are also necessary.

Since theoretical backgrounds on the Hamiltonian and program codes required to deal with these three types of LAMs are available in the two classical books by Gordy and Cook [12] and Townes and Schawlow [95], which were also recently reviewed [2,4,94], the present review will focus on particular molecular examples undergoing these effects: (i) aromatic five-membered rings such as furan, thiophene, pyrrole, thiazole, and oxazole derivatives; (ii) aromatic six-membered rings such as benzene, pyridine, and pyrimidine derivatives; and (iii) larger combined rings such as naphthalene, indole, and indan derivatives. These are sorted into four categories: methyl internal rotation, inversion tunneling, the coupling between these two effects, and ring puckering. For each molecular class, we will present the representatives and summarize the recent insights on the molecular structures and internal dynamics. We explicitly note that only monomers are considered in the present review. Van der Waals complexes undergoing LAMs form a very dynamic, complex, and large topic, and are considered in other reviews.

## 2. Internal Rotation

### 2.1. Monomethyl-Substituted (One-Top) Aromatic Rings

#### 2.1.1. Five-Membered Rings with an (Extended) Conjugated Double-Bond System

The main aromatic five-membered molecular classes mentioned within the frame of the present review are furan, thiophene, oxazole, thiazole, isoxazole, isothiazole, imidazole, and pyrrole.

##### 2.1.1.1. Sole Methyl Substitution on the Ring

**Figure 3:**
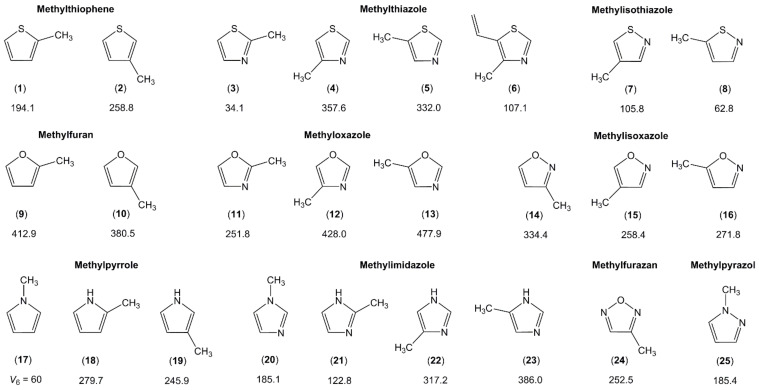
Barriers to methyl internal rotation in monomethyl-substituted planar five-membered rings (in cm^−1^). (**1**) 2-methylthiophene [96], (**2**) 3-methylthiophene [97], (**3**) 2-methylthiazole [98,99], (**4**) 4-methylthiazole [100], (**5**) 5-methylthiazole [101], (**6**) 4-methyl-5-vinylthiazole [102], (**7**) 4-methylisothiazole [103], (**8**) 5-methylisothiazole [104], (**9**) 2-methylfuran [49,105], (**10**) 3-methylfuran [106], (**11**) 2-methyloxazole [107], (**12**) 4-methyloxazole [107,108], (**13**) 5-methyloxazole [107], (**14**) 3-methylisoxazole [109], (**15**) 4-methylisoxazole [110], (**16**) 5-methylisoxazole [109], (**17**) N-methylpyrrole [111], (**18**) 2-methylpyrrole [112], (**19**) 3-methylpyrrole [113], (**20**) N-methylimidazole [114], (**21**) 2-methylimidazole [114], (**22**) 4-methylimidazole [114], (**23**) 5-methylimidazole [114], (**24**) methyl furazan [115], (**25**) N-methylpyrazole [116].

Several classes of monomethyl-substituted planar five-membered rings have been reported in the literature as summarized in Figure 3.

Three pairs can be recognized in the two upper traces: methylfuran and methylthiophene (containing the 2- and 3-isomers), methyloxazole and methylthiazole (containing the 2-, 4-, and 5-isomers), as well as methylisoxazole and methylisothiazole (containing the 3-, 4-, and 5-isomers). Lacking only the study on 3-methylisothiazole, the collection is almost complete. We see a very clear trend that the barrier hindering the methyl torsion is always higher in the oxygen analogues than in the sulfur analogues, i.e., 2-methylthiophene (**1**) (194 cm^−1^) [96] vs. 2-methylfuran (**9**) (413 cm^−1^) [49,105], 4-methylthiazole (**4**) (357 cm^−1^) [100] vs. 4-methyloxazole (**12**) (428 cm^−1^) [107,108], or 5-methylisothiazole (**8**) (63 cm^−1^) [104] vs. 5-methylisoxazole (**16**) (272 cm^−1^) [109]. Note that all given values are experimentally determined. This observation is in agreement with many previous investigations reported in the literature. For example, the two equivalent methyl groups in dimethyl sulfide undergo internal rotations with a torsional barrier of 736 cm^−1^ [117], significantly lower than the value of 944 cm^−1^ found for dimethyl ether [118]. Further examples are trans-ethyl methyl sulfide (693 cm^−1^) [119] vs. trans-ethyl methyl ether (893 cm^−1^) [120], or conformer III of n-propyl sulfide (699 cm^−1^) [121] vs. trans-trans-methyl-n-propyl ether (1154 cm^−1^) [122].

The lowest trace of Figure 3 illustrates the representatives of the methylpyrrole and methylimidazole classes, containing one and two nitrogen nuclei, respectively, in the aromatic ring. In the class of methylpyrrole, the potential of N-methylpyrrole (**17**) possesses a pure V_6_ term with a barrier of about 60 cm^−1^ [111]. The C_2v_ symmetry is broken in the cases of 2- (**18**) and 3-methylpyrrole (**19**), leading to V_3_ potentials of 280 cm^−1^ [112] and 246 cm^−1^ [113], respectively, without significant V_6_ contributions. In methylimidazole, the symmetry is also broken in the case of the N-isomer (**20**) due to the presence of an additional nitrogen atom at the third position of the ring, and only V_3_ potentials were reported for all four isomers of this class [114].

It is difficult to comment on the torsional barriers within each class, since they do not appear to follow any trend. Regarding, for example, the aromatic rings containing only one heteroatom (furan, thiophene, and pyrrole), the barrier to internal rotation of the 2-isomer is lower than that of the 3-isomer in methylthiophene, but the inverse is observed for methylfuran. Similar values are found in 2- and 3-methylpyrrole with that of the 3-isomer being slightly smaller. In aromatic rings with two heteroatoms at the 1 and 3 positions of the ring (oxazole, thiazole, and imidazole), we recognize a trend that the methyl torsional barrier is always higher in the 4- and 5-isomers than in the 2-isomers, and those of methyloxazole are much higher than those of methylthiazole and methylimidazole. An unexpectedly low torsional barrier of only 34 cm^−1^ is found for the methyl group in 2-methylthiazole (**3**) [98,99], while similar values in the intermediate range are observed for the 4- (**4**) and 5-isomers (**5**) [100,101]. The value remains low in 2-methylimidazole (**21**) (123 cm^−1^) [114] and doubles to 252 cm^−1^ in 2-methyloxazole (**11**) [107], but is still the lowest value of the methyloxazole class compared to 428 cm^−1^ in 4-methyloxazole (**12**) [107,108] and 478 cm^−1^ in 5-methyloxazole (**13**) [107]. We notice the remarkably close barriers between the two pairs 4-methylisoxazole (**15**) (258.4 cm^−1^) [110] and methylfurazan (**24**) (252.5 cm^−1^) [115] as well as N-methylimidazole (**20**) (185.1 cm^−1^) [114] and N-methylpyrazole (**25**) (185.4 cm^−1^) [116]. The presence of an additional nitrogen atom in the ring for the former pair as well as the relative positions of the two nitrogen atoms for the latter pair does not seem to affect the methyl torsion.

Steric effects are often cited as the cause of the barrier height of a methyl internal rotation. In cases where steric hindrance effectively does not exist, for example if the internal methyl rotor and the molecular frame are separated by a C≡C-bond, then the torsional barrier is very low, say less than 10 cm^−1^, corresponding to an almost free internal rotation. Some examples are 2-butynoic acid [16], tetrolyl fluoride [123], 1-chloro-2-butyne [124], 3-pentyn-1-ol [125], and 4-hexyn-3-ol [126]. If the methyl group is located in close proximity of an atom or a group of atoms, then the barrier to methyl internal rotation increases to about 400–600 cm^−1^ due steric hindrance, as observed in o-methylanisole [127], 2-halogenotoluene [128], 2-fluoro-4-chlorotoluene [129], 2-chloro-4-fluorotoluene [130], 1,2-dimethylnapthalene [131], 3,4-dimethylfluorobenzene [79], or 3,4-dimethylbenzaldehyde [81]. Predicting whether steric effects occur seems evident, but sometimes the estimation of values for methyl torsional barriers fails because of electronic effects, as in the cases of the 2-isomers of methylthiazole (**3**) [98,99], methyloxazole (**11**) [107], and methylimidazole (**21**) [114] mentioned above. Those methyl groups are sterically more hindered than those in the 4- and the 5-isomers, because an oxygen, a nitrogen, or a sulfur atom is larger than a carbon atom, such that a lower barrier cannot be explained by steric effects, especially in 2-methylthiazole (**3**) with its extremely low barrier of 34 cm^−1^ [98,99]. This can only be explained by bond-mediated electronic interactions. Assuming that a sulfur atom and a nitrogen atom are similar, then the 2-methyl group would experience a six-fold potential arising from a C_2v_ frame symmetry and the C_3v_ symmetry of the methyl group. Then, only a small V_6_ term would exist. But certainly, a sulfur atom and a nitrogen atom are not similar. Therefore, a V_3_ potential is dominant; however, the torsional barrier is still affected, being very low. The same explanation can be made for the oxygen atom and the nitrogen atom in 2-methyloxazole (**11**) [107], or the NH group and the nitrogen atom in 2-methylimidazole (**21**) [114]. Probably, the electronic surroundings of a nitrogen atom is more similar to that of a sulfur atom than that of an NH group or an oxygen atom. While lacking conclusive reasoning, e.g., steric effects, for this statement, there is a high probability to observe unexpectedly low torsional barriers if a methyl group is squashed between two groups/atoms.

##### 2.1.1.2. Carbonyl Substituent on the Ring

**Figure 4:**
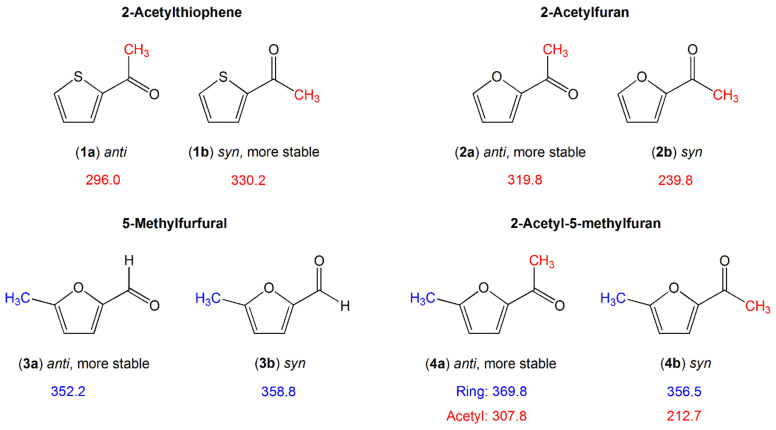
Barriers to methyl internal rotation in furan and thiophene derivatives with a carbonyl-group-containing substituent (in cm^−1^). (**1**) 2-Acetylthiophene [132], (**2**) 2-acetylfuran [133], (**3**) 5-methyl furfural [134], (**4**) 2-acetyl-5-methylfurfural [86].

There are also investigations of molecules in which a carbonyl-group-containing substituent (a formyl or an acetyl group) is present, as summarized in Figure 4. 

The conjugated π-double-bond system is extended by the carbonyl group C=O. The methyl group can be directly attached to the aromatic ring in the case of a formyl substituent as in 5-methyl furfural (**3**) [134], or be involved in the substituted moiety in the case of an acetyl substituent such as in acetyl thiophene (**1**) [132] or acetyl furane (**2**) [133]. A two-top molecule is also given in this section, 2-acetyl-5-methylfuran (**4**) [86], combining a methyl rotor attached on the aromatic ring as in 5-methyl furfural (**3**) and a methyl rotor in the acetyl moiety as in acetyl furan (**2**) for comparison.

The comparison within the furan class clearly illustrates the dependence of the methyl torsion on electronic interactions which expand over the aromatic system. The presence of a carbonyl substituent with its negative mesomeric and inductive effects notably decreases the torsional barrier of the ring methyl group of 2-methylfuran (412.9 cm^−1^, molecule (**9**) in Figure 3) [49,105] to about 360 cm^−1^ in both 5-methyl furfural (**3**) [134] and 2-acetyl-5-methylfuran (**4**) [86]. The barrier height of the acetyl methyl group in syn- and anti-2-acetylfuran (**2**) [133] is higher than that of the corresponding conformer of 2-acetyl-5-methylfuran (**4**) [86]. Note that syn and anti are used to define the orientation of the carbonyl group relative to the heteroatom in the ring. The only structural difference between the two molecules is the addition of a methyl group on the furan ring, making the moiety connected to the acetyl group longer. Andresen et al. reported on the so-called chain-length effects observed for linear aliphatic ketones containing an acetyl group. The torsional barrier of the acetyl methyl group is lower in ketones with a longer alkyl chain [135]. The same effects might explain the torsional barrier of 2-acetyl-5-methylfuran (**4**) [86] being lower than that of 2-acetylfuran (**2**) [133]. The difference is more pronounced for the syn-conformers (240 cm^−1^ in 2-acetylfuran vs. 213 cm^−1^ in 2-acetyl-5-methylfuran) than for the anti-conformers (320 cm^−1^ vs. 308 cm^−1^, respectively), because for the former, the methyl group “senses” a longer substituent when a methyl group is added at the fifth position of the ring than it does in the anti-conformation.

The barrier height of the ring methyl group in 5-methylfurfural (**3**) and 2-acetyl-5-methylfuran (**4**) is almost conformational independent. This is not the case for the acetyl methyl group. The value of 240 cm^−1^ of the less stable syn-conformer of 2-acetylfuran (**2b**) is significantly lower than that of the anti-conformer (**2a**), which is also the observation found for 2-acetyl-5-methylfuran (**4**). In 2-acetylthiophene (**1**), the syn-conformer is more stable and possesses a higher methyl torsional barrier. The more stable conformer seems to feature a higher barrier. Since the conformational stability in these carbonyl-group-containing five-membered aromatic rings is connected to the charge distribution within the molecule [136], this supports the hypothesis that electronic interaction plays an important role in the acetyl methyl torsion.

Comparing the barrier height of 2-acetylthiophene (**1**) and 2-acetylfuran (**2**), we surprisingly find that for the syn-conformers, the higher torsional barrier is observed for the sulfur analogue, in contrast to the frequent observation that the torsional barriers of methyl internal rotors in oxygen analogues are higher, which remains true for the anti-conformer. We have no explanation for this rather surprising observation, but again suspect the electronic effects to be the reason.

#### 2.1.2. Six-Membered Rings with an (Extended) Conjugated Double-Bond System

The main aromatic six-membered molecular classes considered within the frame of the present review are benzene, pyridine, and pyrimidine. The prototype of all benzene derivatives with one methyl internal rotor, toluene, forms its own class with many previous investigations and will not be given in full detail here. We will only briefly summarize some studies on toluene and compare its torsional barrier, being a pure V_6_ potential due to the C_2v_ symmetry of the C_6_H_5_ ring in combination with the C_3v_ symmetry of the methyl group, with that of other derivatives. Concerning electronic effects, the methyl top reflects its local electronic surroundings, serving as an exquisite probe, and fascinating insights are provided by the methyl-internal-rotation potentials. With further substituents added to the ring, the pure V_6_ potential of toluene varies strongly in shape and height. The C_2v_ symmetry of the phenyl ring is often broken, and a V_3_ term occurs.

##### 2.1.2.1. Toluene

This prolate top with a Ray’s asymmetry parameter of −0.59 and an almost free, sixfold barrier methyl internal rotation has been studied in the microwave range for four decades. Starting with the work of Rudolph et al. in 1967, where some A-symmetry-species transitions of low J-values in the torsional ground states, labeled m = 0 ($A_{1}^{\prime }$) and m = 3 ($A_{2}^{\prime }$) in the free-rotor basis, which is appropriate for this very-low-barrier case, were assigned, the dipole-moment components were measured, and a V_6_ term of 4.88(3) cm^−1^ was deduced [137]. Later on, the spectra of ^13^C and deuterated isotopologues were also recorded with the aim of determining the toluene structure [138,139]. New results on the microwave spectrum of toluene were reported in 2004 by Kisiel et al. in the frequency range from 3.5 GHz to 26.5 GHz, supplemented by the millimeter-wave range of 160 to 330 GHz [140]. Extensive work on toluene was then performed by Ilyushin et al. [141], where the gap between 49 GHz to 160 GHz was closed in 2017 using the Kharkov and Warsaw spectrometers [142]. The most recent data set of toluene covers the 3.5 to 26.6 GHz and 49–336 GHz spectral ranges and contains rotational transitions with *J* ≤ 94, *K_a_* ≤ 50, and m = 0, 1, 2, ±3 states [142].

##### 2.1.2.2. Cresol and Methylanisole Derivatives

**Figure 5:**
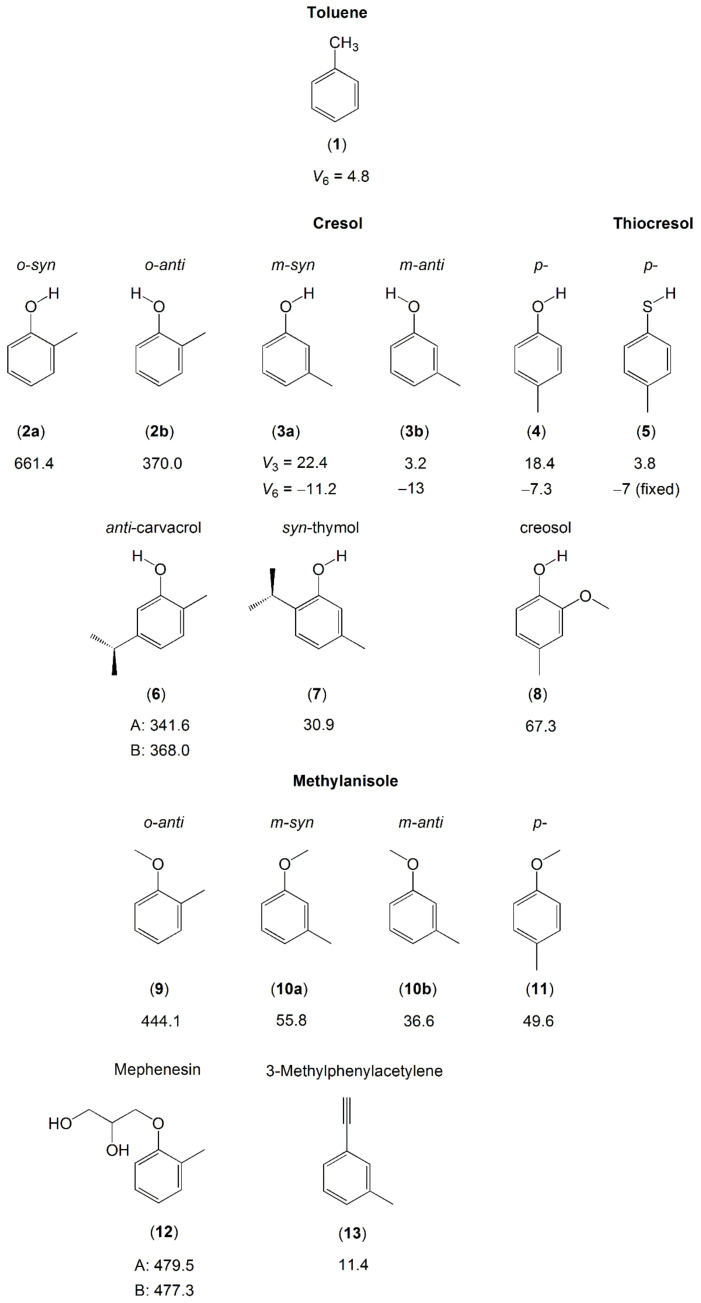
Barriers to methyl internal rotation in toluene and cresol derivatives (in cm^−1^). (**1**) The prototype toluene [137,138,139,140,141,142], (**2**) o-cresol [143], (**3**) m-cresol [144], (**4**) p-cresol [145], (**5**) p-thiocresol [146], (**6**) anti-carvacrol (two conformers, A and B) [147], (**7**) syn-thymol [147], (**8**) creosol [148], (**9**) o-methyl anisole [127], (**10**) m-methyl anisole [149,150], (**11**) p-methylanisole [151], (**12**) mephenesin (two conformers, A and B) [152], (**13**) 3-methylphenylacetylene [153].

Cresol and methylanisole are toluene derivatives in which a hydroxyl OH or a methoxy O-CH_3_ substitution, respectively, is attached to the ring at three different positions related to the methyl group—ortho, meta, and para— resulting in three structural isomers. Although some chemical properties such as vapor pressure, color, smell, and acidity are similar, the effects of methyl internal rotation, which often depend on the steric and electronic surroundings, are completely different for these isomers, as shown in Figure 5.

While only one conformer exists for p-cresol (**4**) [145] and p-methylanisole (**11**) [151] due to symmetry, two conformers of m-cresol (**3**) [144] and m-methylanisole (**10**) [149] with the OH or the methoxy group in an anti or a syn position were observed in the microwave spectra. The conformers possess quite different torsional barriers of the ring methyl group. For o-cresol (**2**), Welzel et al. also reported on both the syn- and the anti-conformers [143]. Due to steric hindrance arising from the bulkier methoxy group, only the anti-conformer of o-methylanisole (**9**) [127] was observed; the syn-conformer was too high in energy, similar to the situation found for mephenesin (**12**) [152].

Comparing the barrier heights of all three isomers in the cresol and methylanisole families, the V_3_ potential changes for each isomer because the methyl rotor encounters different local environments. We find a clear trend that the torsional barrier of the o-isomer is largest. The barrier height is dominated by steric hindrance, since the substituents are adjacent to each other on the benzene ring. The barriers decrease in the m-isomers, because the substituents are further apart, which create a symmetric local environment near the methyl group, even though the global frame of the molecule is still asymmetric. The p-isomers also possess a very small barrier, because the molecules are electronically and structurally symmetric. This trend also occurs in the cresol derivatives carvacrol (**6**), thymol (**7**) [147], and creosol (**8**) [148], as well as in 3-methylphenylacetylene (**13**) [153]. Note that the torsional barrier of the methyl group in the methoxy moiety of methylanisole and those of the two methyl groups in the isopropyl moiety of carvacrol and thymol are high. Therefore, no splittings arising from these methyl torsions are observed.

Regarding within the cresol or the methylanisole family, we provide some comments on the conformational effects on methyl torsional barriers. The studies on o-cresol (**2**) [143], m-cresol (**3**) [144], and m-methylanisole (**10**) [149] have explored significant differences in the V_3_ potentials between the rotational conformers, where in all cases, the barrier for the anti-conformer is lower. As mentioned above, there are two factors that affect the height of a methyl torsional barrier: steric hindrance and electronic configuration. For the o-isomers, steric effects are clearly responsible for the lower barriers in the anti-configuration. For the m-isomers, though the barriers found in the anti-conformers are still significantly lower, it is unlikely that steric effects are the reason because of the great distance between the two substituents. This implies that electronic properties are more likely responsible for the different barrier heights.

Very similar barriers to methyl internal rotation are found for the anti-conformers of o-cresol (**2b**) (370 cm^−1^) [143] and carvacrol (**6**) (342 cm^−1^ for conformer A and 368 cm^−1^ for conformer B) [147], showing that the presence of an isopropyl group at the other side of the phenyl ring does not affect the methyl torsion. The barrier height of the o-methyl group in anti-o-methylanisole (**9**) [127] and mephenesin (**12**) [152] also remains almost unchanged, independent on the length of the O-R substitution.

##### 2.1.2.3. Carbonyl Substituent on the Ring

**Figure 6:**
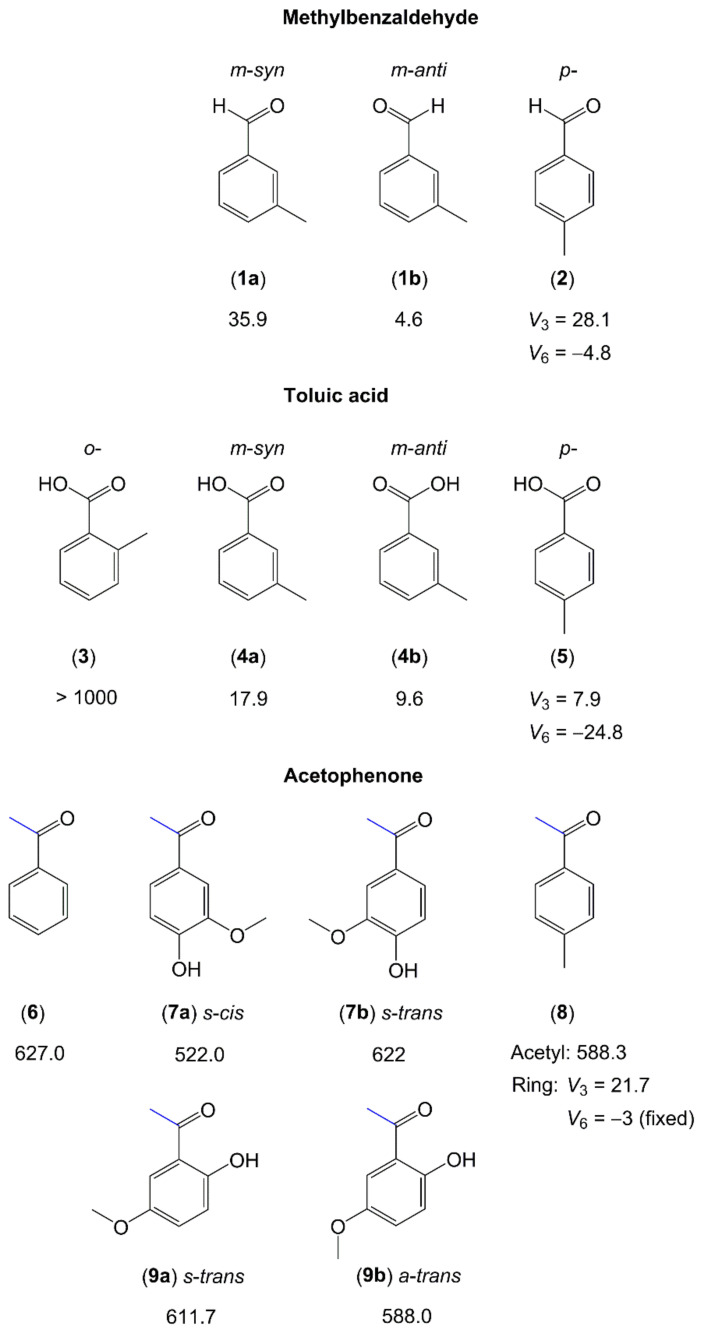
Barriers to methyl internal rotation in toluene derivatives with a carbonyl-containing substituent (in cm^−1^). (**1**) m-Tolualdehyde [154], (**2**) p-tolualdehyde [155], (**3**) o-toluic acid [156], (**4**) m-toluic acid [157], (**5**) p-toluic acid [65], (**6**) acetophenone [158], (**7**) acetovanillone [159], (**8**) 4-methylacetophenone [77,160], (**9**) 6-hydroxy-3-methoxyacetophenone [159].

Similar to the case of aromatic five-membered rings, several six-membered rings containing a carbonyl group that extends the conjugated π-double-bond system were systematically investigated by microwave spectroscopy. The representatives are methyl benzaldehyde, toluic acid, acetophenone and its derivatives. Figure 6 summarizes their experimental methyl torsional barriers.

Resembling cresols and methylanisoles (see Section 2.1.2.2 and Figure 5), microwave studies on three isomers of toluic acid have shown that the V_3_ potential is so high in the o-isomer (molecule (**3**) in Figure 6) that no torsional splittings could be observed [156]. The barrier to methyl internal rotation drastically decreases in the meta- [157] and para-isomers [65]. m-toluic acid (**4**) [157] and m-methylbenzaldehyde (**1**) [154] both exist in two configurations in the microwave spectrum, syn and anti, where the anti-conformers feature lower methyl torsional barriers. Substitution at the para position of toluene produces a low barrier to methyl torsion, which is confirmed in the case of p-toluic acid (**5**) with a V_3_ term of 7.899(1) cm^−1^ and a V_6_ leading term of −24.77(2) cm^−1^ in the potential [65]. For p-tolualdehyde (**2**), a V_3_ potential of 28 cm^−1^ with a V_6_ contribution of −4.8 cm^−1^ [155] is reported. In the investigations of 4-methylacetophenone (**8**) [77,160], the value of approximately 22 cm^−1^ of the p-methyl rotor is close to the value of 18 cm^−1^ observed for p-cresol (molecule (**4**) in Figure 5) [145] and 28 cm^−1^ for p-tolualdehyde (**2**), but much lower than the value of 50 cm^−1^ of p-methylanisole (molecule (**11**) in Figure 5) [151]. If substituting the para position of toluene breaks the C_2v_ symmetry of toluene less effectively in terms of symmetry-breaking electronic contributions, then a smaller V_3_ contribution is obtained. The COOH group in p-toluic acid (**5**) is almost C_2v_ symmetric, making the V_6_ contribution the leading term, which is much larger than V_3_. In p-cresol, p-tolualdehyde (**2**), and 4-methylacetophenone (**8**), the substituent at the para position of toluene is more C_2v_-symmetric than that in p-methylanisole (OH ≈ O=C−CH_3_ ≈ CHO < O−CH_3_). The observed V_3_ term of the para methyl group in p-methylanisole is consequently larger (18 cm^−1^ ≈ 22 cm^−1^ ≈ 28 cm^−1^ < 50 cm^−1^).

For molecules containing an acetyl group, the methyl internal rotor is involved in the acetyl moiety and a phenyl ring is attached to the other side of the carbonyl group. The acetyl methyl torsion is found to be 627 cm^−1^ in acetophenone (**6**) [158], which is the simplest ketone containing an acetyl group attached to a phenyl ring. In two derivatives of acetophenone, acetovanillone (**7**) and 6-hydroxy-3-methoxyacetophenone (**9**), which are isomers, a value between 522 and 622 cm^−1^ is observed for the torsional barrier, depending on the respective conformer [159], close to the value of 588 cm^−1^ found for the acetyl methyl group of 4-methylacetophenone (**8**) [77]. This leads to the conclusion that the acetyl methyl torsional barrier is approximately 600 cm^−1^ if a phenyl ring is attached directly to the other side of the carbonyl group. For the methoxy methyl group of acetovanillone (**7**) and 6-hydroxy-3-methoxyacetophenone (**9**), no splittings arising from the methyl torsion are observed, similar to the cases of methylanisoles (molecules **9**–**11** in Figure 5).

##### 2.1.2.4. Halogen Substituent(s) on the Ring

**Figure 7:**
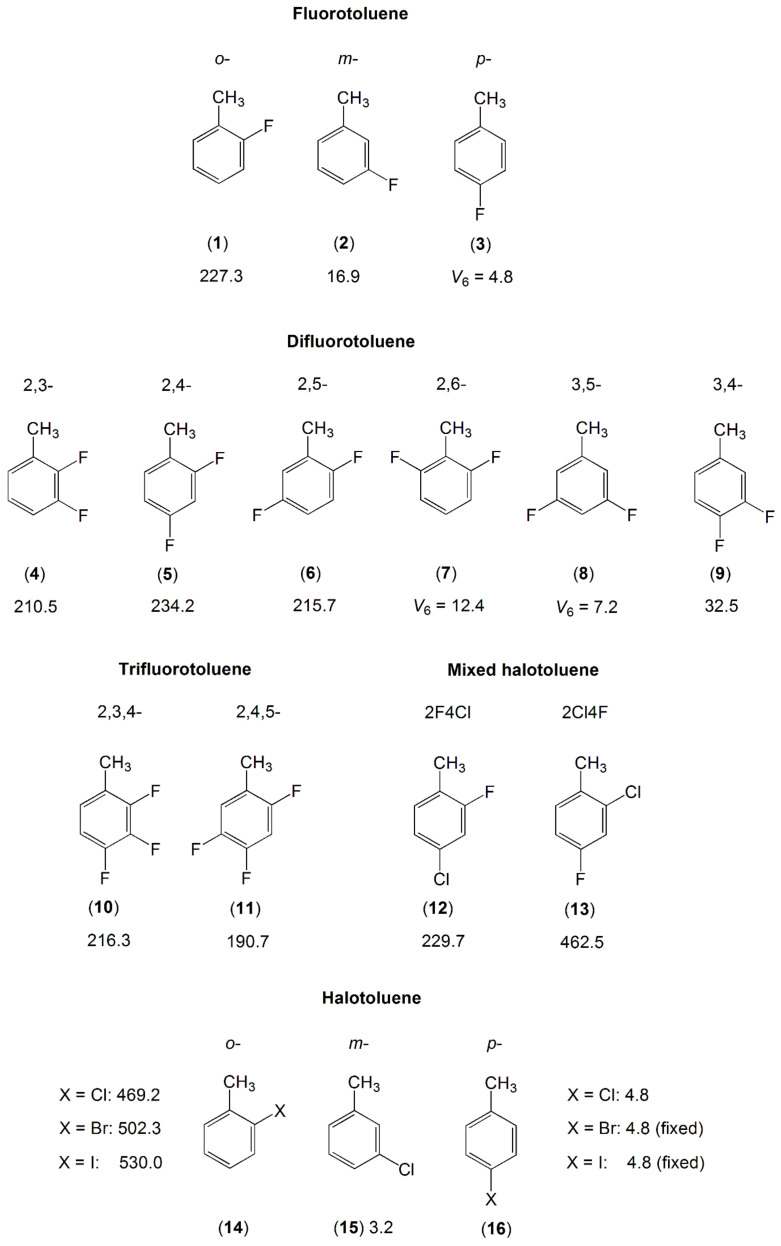
Barriers to methyl internal rotation in halogen-substituted toluene derivatives (in cm^−1^). (**1**) o-fluorotoluene [161], (**2**) m-fluorotoluene [162,163], (**3**) p-fluorotoluene [164], (**4**) 2,3-difluorotoluene [165], (**5**) 2,4-difluorotoluene [166], (**6**) 2,5-difluorotoluene [167], (**7**) 2,6-difluorotoluene [168], (**8**) 3,5-difluorotoluene [168], (**9**) 3,4-difluorotoluene [163,169], (**10**) 2,3,4-trifluorotoluene [170], (**11**) 2,4,5-trifluorotoluene [170], (**12**) 2-fluoro-4-chlorotoluene [129], (**13**) 2-chloro-4-fluorotoluene [130], (**14**) o-X-toluene [128], (**15**) m-chlorotoluene [171], (**16**) p-X-toluene [172], X = Cl, Br, I.

The fluoro-substituted toluene derivatives form a large body with many previous investigations, for example the fluorotoluene family with its three isomers [161,162,163,164], the difluorotoluenes with six isomers [165,166,167,168,169], and trifluorotoluenes with two isomers [170] are reported in the literature. The number of investigations on chloro-substituted toluenes is smaller and limited to the three isomers ortho- [128], meta- [171], and para-chlorotoluene [172] and the two mixed halogen-substituted toluenes, 2-fluoro-4-chlorotoluene [129] and 2-chloro-4-fluorotoluene [130]. For the o- and p-isomers, studies on bromo- and iodotoluene also exist [128,172]. The molecules are illustrated in Figure 7.

Regarding two complete series of mono-halotoluenes X-C_6_H_4_-CH_3_ with X = F and Cl, the V_3_ potentials of 17 cm^−1^ and 3.2 cm^−1^ found for 3-fluorotoluene (**2**) [163] and 3-chlorotoluene (**15**) [171] are extremely low. From the molecules illustrated in Figure 7, except those with a pure V_6_ potential due to symmetry (the p-isomers, 2,6- and 3,5-dimethylfluorobenzene), if the methyl group is free of a neighbor substituent, then the torsional barrier is generally lower than 50 cm^−1^. This is also the case for 3,4-difluorotoluene (**9**) [163,169] as well as all meta-mono-substituted toluenes previously mentioned such as m-methylanisole (molecule (**10**) in Figure 5, 55.8 and 36.6 cm^−1^ for the syn- and anti-conformers, respectively) [149,150], m-cresol (molecule (**3**) in Figure 5, syn: 22.4 cm^−1^, anti: 3.2 cm^−1^) [144], and m-tolualdehyde (molecule (**1**) in Figure 6, syn: 35.9 cm^−1^, anti: 4.6 cm^−1^) [154]. If a halogen atom is at a substitution position next to the methyl group, causing steric hindrance of the methyl torsion, then the barrier to internal rotation becomes intermediate. 

A comparison of the V_3_ potential terms of 2-fluoro substituted toluenes (**1**), (**4**–**6**), (**10**–**12**) shows that just a single fluorine atom in the immediate neighborhood of the methyl group leaves the barrier hindering the methyl torsion largely invariant at a value around 230 cm^−1^ [129,161,165,166,167,170]. Further substitutions on the ring do not affect the barrier height significantly. However, if the ortho fluorine atom is exchanged by a bulkier chlorine atom, as in the cases of 2-chloro-4-fluorotoluene (**13**) [130] and 2-chlorotoluene (**14**) [128], then the barrier to internal rotation of the methyl group almost doubles due to steric effects. The larger the halogen atom, the higher the barrier, as can be seen in the o-halogen toluenes with the halogen atom being F, Cl, Br, and I [128,161]. The p-halogen toluenes (**16**) [172] as well as 2,6- (**7**) and 3,5-difluorotoluene (**8**) [168] experience a low pure V_6_ barrier similar to that of toluene (molecule (**1**) in Figure 5) [137,138,139,140,141,142].

##### 2.1.2.5. Nitrogen-Containing Aromatic Six-Membered Ring

**Figure 8:**
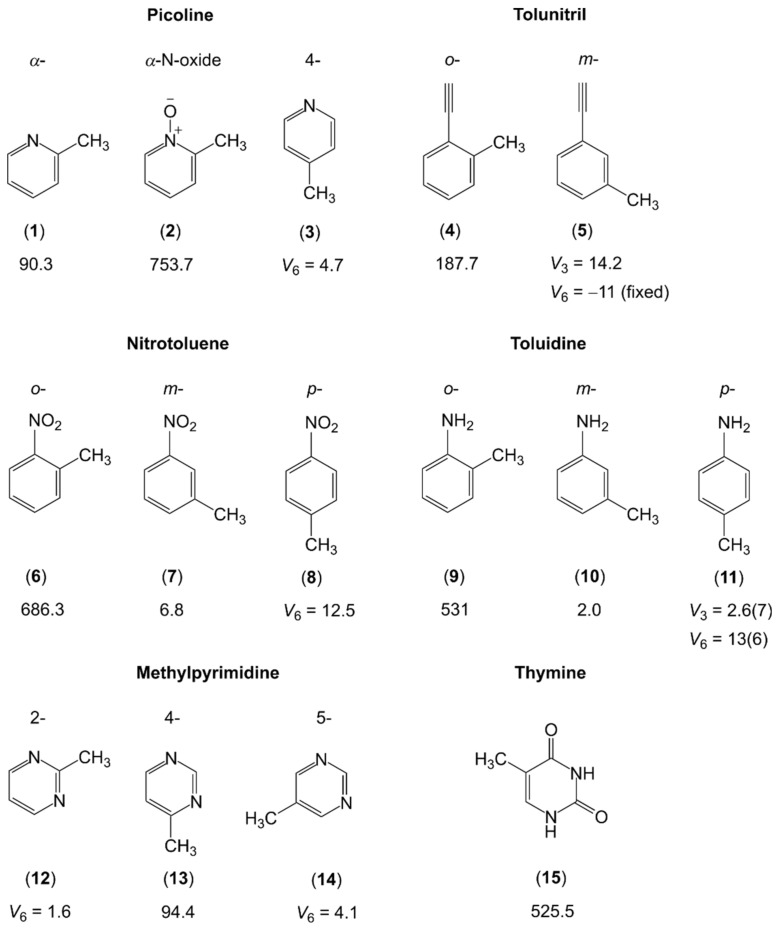
Barriers to methyl internal rotation in nitrogen-containing aromatic six-membered ring (in cm^−1^). (**1**) α-picoline [173,174], (**2**) α-picoline-N-oxide [175], (**3**) 4-picoline [176], (**4**) o-tolunitril [177,178], (**5**) m-tolunitril [179], (**6**) o-nitrotoluene [180], (**7**) m-nitrotoluene [181], (**8**) p-nitrotoluene [180], (**9**) o-toluidine [182], (**10**) m-toluidine [182], (**11**) p-toluidine [183], (**12**) 2-methylpyrimidine [184], (**13**) 4-methylpyrimidine [185], (**14**) 5-methylpyrimidine [186], (**15**) thymine [187].

The investigations of monomethyl-substituted aromatic six-membered ring containing a nitrogen atom are limited to α-picoline [173,174], its N-oxide derivative [175], and 4-picoline [176], the two isomers (ortho and meta) of tolunitril [177,178,179], as well as the three isomers of nitrotoluene [180,181] and toluidine [182,183]. This class of compounds also includes the methylpyrimidine family and thymine, in which the microwave spectra are significantly complicated by the presence of two nitrogen nuclei [184,185,186,187]. The molecules are illustrated in Figure 8.

Though limited in the number of available studies, steric effects are well-reflected. If only the nitrogen atom of the aromatic ring or a linear group of atoms such as the cyano group is in the neighborhood of the methyl rotor, then the torsional barriers are low, e.g., 90.3 cm^−1^ for α-picoline (**1**) [173,174], which is essentially the same as 94.4 cm^−1^ for 4-methylpyrimidine (**13**) [185], while the value of 187.7 cm^−1^ for o-tolunitril (**4**) [177,178] is still rather low. The value increases tremendously to 525.5 cm^−1^ in thymine (**15**) [187], 531 cm^−1^ in o-toluidine (**9**) [182], and 686.3 cm^−1^ in o-nitrotoluene (**6**) [180]. These values are significantly higher than that of 444 cm^−1^ found for o-methylanisole (molecule (**9**) in Figure 5) [127]. In α-picoline-N-oxide (**2**), Heineking et al. reported on an even higher value of 753.7 cm^−1^ [175]. The steric hindrance for the methyl rotor should be the same in α-picoline-N-oxide as it is in o-methylanisole. Therefore, electrostatic interactions caused by pronounced partial charges on the oxygen and the nitrogen atoms might be the cause of the remarkable difference in barrier heights between the two molecules. We observe the same trend as in Figure 6, that the torsional barriers of the m- and p-isomers and, if induced by symmetry, V_6_ potentials are very low. Interestingly, the V_6_ potential of p-nitrotoluene (**8**) [180] is higher than the V_3_ potential of the m-isomer (**7**) [181], and a surprisingly low V_3_ barrier of only 2.0 cm^−1^ is found for m-toluidine (**10**) [182].

#### 2.1.3. Larger Methyl-Substituted Aromatic Rings

**Figure 9:**
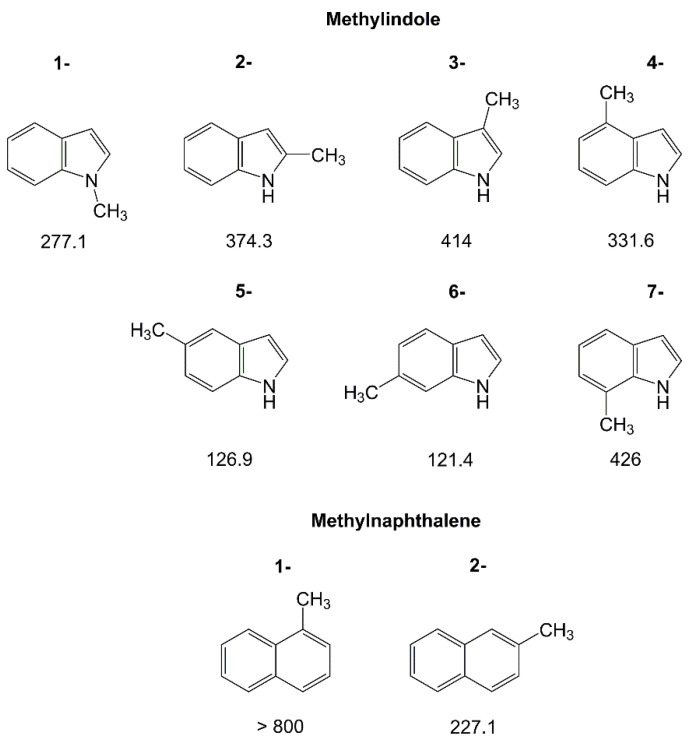
Barriers to methyl internal rotation in large aromatic rings (in cm^−1^). (**1**) 1-Methylindole [188], (**2**) 2-methylindole [188], (**3**) 3-methylindole [188], (**4**) 4-methylindole [188], (**5**) 5-methylindole [188], (**6**) 6-methylindole [188], (**7**) 7-methylindole [188], (**8**) 1-methylnaphthalene [131], (**9**) 2-methylnaphthalene [131].

Due to the low vapor pressure, the number of studied aromatic rings larger than six-membered with methyl internal rotation is very limited. Currently, there are only investigations of methylnaphthalenes [131] and the seven isomers of methylindole [188], visualized in Figure 9. In such large rings, the torsional barrier of the methyl top reflects well the electronic environment around the perimeter of the ring. Regarding the methylindoles, e.g., the lowest barriers are found for the 5- and 6-isomers, which are very similar (126.9 cm^−1^ and 121.4 cm^−1^, respectively). The two methyl groups experience almost the same steric and electronic surroundings from their positions attached to the ring. The highest barriers are those of the 3- and the 7-isomers. With values of 414 cm^−1^ and 426 cm^−1^, respectively, they are also very similar, though the two methyl groups are attached to different parts of the indole ring. Note that both methyl groups are separated, while still electronically connected through the π-system, by two carbon atoms from the nitrogen atom. The intermediate barrier heights of 374.3 cm^−1^ and 331.6 cm^−1^ of 2-methylindole and 4-methylindole, respectively, lay in between. 1-methylindole distinguishes itself from the other isomers in the family from the methyl substitution at the nitrogen atom, with its intermediate torsional barrier of 277.1 cm^−1^. Gurusinghe and Tubergen associated this value to the 0.05 Å shortened rotor length due to the higher electronegativity of the nitrogen atom, but also to two localized molecular-orbital single bonds in its vicinity with the largest contributions being nonlocal-bonding–anti-bonding interactions of two localized molecular-orbital C–C bonds on either side of to the rotor [188].

Turning to methylnaphthalene, the high symmetry of the naphthalene ring only allows for two isomers, 1-methylnaphthalene and 2-methylnaphthalene. For the 1-isomer, no splittings are observed in the spectrum due to methyl internal rotation and therefore, the barrier height cannot be determined [131]. This corresponds to a high value, in agreement with the results obtained by Tan et al. from rotationally resolved electronic spectroscopy which reported a barrier of 811 cm^−1^ [189]. The value of 227.1 cm^−1^ found for 2-methylnaphthalene is drastically lower. The local steric surroundings of the methyl group in the two isomers are similar, such that such a decrease in barrier height from 1-methylnaphthalene to 2-methylnaphthalene is most probably of electronic origin.

### 2.2. Dimethyl-Substituted Aromatic Rings

This section deals with aromatic rings with two methyl internal rotors. While a vast number of studies on one-top problems are available for the previous Section 2.1, the number drastically decreases for the two-top cases. However, we try to keep the section details similar to those of Section 2.1.

#### 2.2.1. Dimethyl-Substituted Five-Membered Rings

##### Sole Dimethyl Substitutions on the Ring

**Figure 10:**
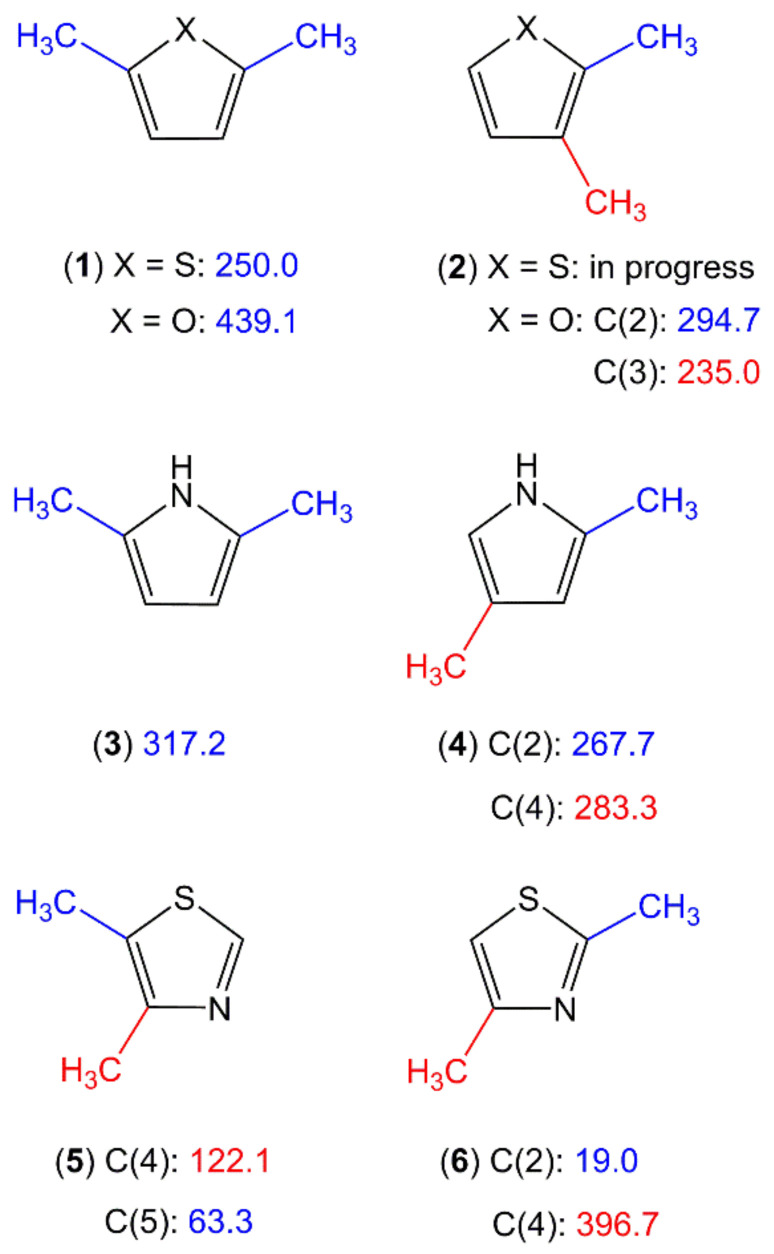
Torsional barriers of the ring methyl rotors in dimethyl-substituted planar five-membered rings (in cm^−1^). (**1**) X = O: 2,5-dimethylfuran [88], X = S: 2,5-dimethylthiophene [89], (**2**) X = O: 2,3-dimethylfuran [190], X = S: 2,3-dimethylthiophene, (**3**) 2,5-dimethylpyrrole [87], (**4**) 2,4-dimethylpyrrole [191], (**5**) 4,5-dimethylthiazole [85], and (**6**) 2,4-dimethylthiazole [192].

Only four dimethyl-substituted planar five-member rings, 4,5-dimethylthiazole [85], 2,5-dimethylpyrrole [87], 2,5-dimethylfuran [88], and 2,5-dimethylthiophene [89], are published in the literature. Studies in progress include the 2,3-isomers of dimethylfuran [190] and dimethylthiophene as well as the 2,4-isomers of dimethylpyrrole [191] and dimethylthiazole [192]. Figure 10 provides an overview of this class of molecules. With these few data points, it is not possible to draw a trend of the methyl torsional barriers. We observed for the methyl group at the 2-position of 2,4-dimethylthiazole (**6**) an unexpectedly low barrier of 19.1 cm^−1^ [192], the same situation as that of 2-methylthiazole (molecule (**3**) in Figure 3) [98,99]. Though slightly higher, the values of 122.1 cm^−1^ and 63.3 cm^−1^ found for the 4- and the 5-methyl groups, respectively, of 4,5-dimethylthiazole (**5**) [85] are still too low to be explained by steric effects, especially if compared to the intermediate value of 396.7 observed for the 4-methyl group of the 2,4-isomer (**6**), as well as those of the dimethylfurane (**1**, **2**) [88,190], dimethylthiophene (**1**, **2**) [89], and dimethyl-pyrrole (**3**, **4**) [87,191] families. This again hints at electronic effects as the reason.

##### Methyl Substitution on a Substituent

**Figure 11:**
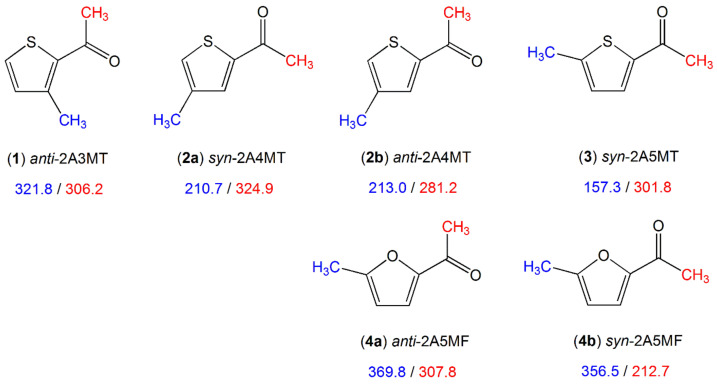
Barriers to methyl internal rotation in furan and thiophene derivatives with a methyl rotor on the ring and an acetyl group which contains another methyl rotor (in cm^−1^). (**1**) anti-2-acetyl-3-methylthiophene [193], (**2**) 2-acetyl-4-methylthiophene [194], (**3**) syn-2-acetyl-5-methylthiophene [195], (**4**) 2-acetyl-5-methylfuran [86].

The furan and thiophene derivatives illustrated in Figure 11 all possess a methyl group attached to the ring and an acetyl methyl group.

A comparison of the ring methyl barrier in syn-2-acetyl-5-methylthiophene (**3**) (157.3 cm^−1^) [195] to that of 2-methylthiophene (molecule (**1**) in Figure 3, 194.1 cm^−1^) [96] and 2,5-dimethylthiophene (molecule (**1**) in Figure 10, 250.0 cm^−1^) [89] as well as the ring methyl barrier in 2-acetyl-5-methylfuran (**4**) (syn 356.5 cm^−1^, anti 369.8 cm^−1^) [86] to that of 2-methylfuran (molecule (**9**) in Figure 3, 412.9 cm^−1^) [105] and 2,5-dimethylfuran (molecule (**1**) in Figure 10, 439.1 cm^−1^) [88] demonstrates that different substituents possess different electronic effects. The carbonyl moiety in the acetyl groups with its negative mesomeric and inductive effects causes a decrease in the torsional barrier, and a methyl group causes an increase when substituted into the same position due to its positive inductive effect. The only case where the addition of the acetyl group leads to an increase in the barrier to methyl internal rotation is anti-2-acetyl-3-methylthiophene (**1**) (321.8 cm^−1^) [193], compared to 3-methylthiophene (258.8 cm^−1^, molecule (**2**) in Figure 3) [97], due to steric hindrance between the methyl group attached to the ring and the acetyl group. The orientation of the acetyl group has only negligible influences on the barrier of the ring methyl rotor as can be seen in the cases of syn-2-acetyl-4-methylthiophene (**2a**) (210.7 cm^−1^) and anti-2-acetyl-4-methylthiophene (**2b**) (213.0 cm^−1^) [194].

**Figure 12:**
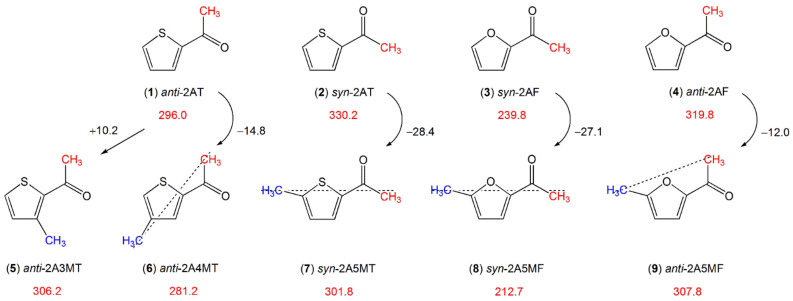
Barriers to methyl internal rotation in furan and thiophene derivatives with an acetyl-group-containing substituent (in cm^−1^). (**1**) anti-2-acetylthiophene [132], (**2**) syn-2-acetylthiophene [132], (**3**) syn-2-acetylfuran [133], (**4**) anti-2-acetylfuran [133], (**5**) anti-2-acetyl-3-methylthiophene [193], (**6**) anti-2-acetyl-4-methylthiophene [194], (**7**) syn-2-acetyl-5-methylthiophene [195], (**8**) syn-2-acetyl-5-methylfuran [86], (**9**) anti-2-acetyl-5-methylfuran [86].

The torsional barrier of the acetyl methyl rotor is also affected by the ring methyl group. Starting with the values of 296.0 cm^−1^ for anti- (**1**) and 330.2 cm^−1^ for syn-2-acetylthiophene (**2**) (see Figure 12), adding a methyl group at the 4- (2-acetyl-4-methylthiophene, (**6**)) or the 5-position (2-acetyl-5-methylthiophene, (**7**)) of the thiophene ring decreases the barrier, but adding the methyl group at the 3-position (2-acetyl-3-methylthiophene, (**5**)) increases it. This can again be explained by the so-called chain-length effect reported for linear aliphatic ketones containing an acetyl group [135]. Ketones with longer chains feature more prolate character and the acetyl methyl groups possess lower torsional barriers. The effect is reported to be stronger for linear aliphatic ketones with a C_s_ structure and thus a “straighter” chain than those with a C_1_ structure and a “bent” chain. 

We found the same situation while comparing the molecules in Figure 12. In anti-2-acetyl-4-methylthiophene (**6**) [194] (−14.8 cm^−1^ compared to anti-2-acetylthiophene (**1**) [132]), syn-2-acetyl-5-methylthiophene (**7**) [195] (−28.4 cm^−1^ compared to syn-2-acetylthiophene (**2**) [132]), syn-2-acetyl-5-methylfuran (**8**) [86] (−27.1 cm^−1^ compared to syn-2-acetylfuran (**3**) [133]), the acetyl methyl and ring methyl group lie on a straight chain (marked by a dotted line in Figure 12), and the torsional barrier of the acetyl methyl rotor decreases more than it does for anti-2-acetyl-5-methylfuran (**9**) (−12.0 cm^−1^ compared to anti-2-acetylfuran (**4**)). In anti-2-acetyl-3-methylthiophene (**5**) [193], the additional methyl group at the 3-position of the ring makes the molecule more globular than anti-2-acetylthiophene (**1**). Therefore, the barrier hindering the acetyl methyl torsion increases.

#### 2.2.2. Dimethyl-Substituted Six-Membered Rings

**Figure 13:**
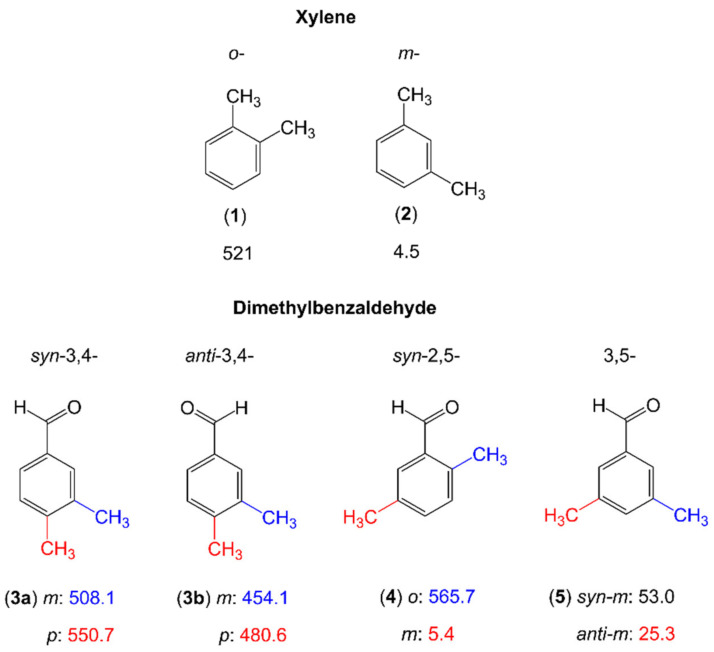
Barriers to methyl internal rotation in xylenes and dimethylbenzaldehydes (in cm^−1^). (**1**) o-xylene [196], (**2**) m-xylene [197], (**3**) 3,4-dimethylbenzaldehyde [81], (**4**) 2,5-dimethyl-benzaldehyde [81], 3,5-dimethylbenzaldehyde [81].

In only a few cases reported in the literature, such as the three isomers of dimethylbenzaldehyde [81] and two isomers of xylene (*o*- and *m*-) [196,197], the methyl rotors are attached to a phenyl ring (see Figure 13). We recognize a clear trend that the barrier height is extremely low (around 5 cm^−1^) for the meta methyl groups of m-xylene (**2**) [197] and syn-2,5-dimethylbenzaldehyde (**4**) [81] due to the absence of steric hinderance. While still being very low, the values found for the two meta methyl groups of 3,5-dimethylbenzaldehyde (**5**) [81] are an order of magnitude larger. This demonstrates again that not only steric hindrance, but also the electronic environment plays an important role in the methyl torsions. In o-xylene (**1**), the 3,4-isomer (**3**) as well as the o-methyl group of the 2,5-isomer (**4**) of dimethylbenzaldehyde, the methyl internal rotation barriers are intermediate, ranging from about 450 cm^−1^ to about 560 cm^−1^, attributed to steric reason. Currently, several studies are focusing on two dimethylbenzene families, dimethylanisole and dimethylfluorobenzene, aiming at studying a complete series of molecules in order to understand the methyl torsions around the ring perimeter.

##### 2.2.2.1. Coupled Internal Rotations in Dimethylanisoles

**Figure 14:**
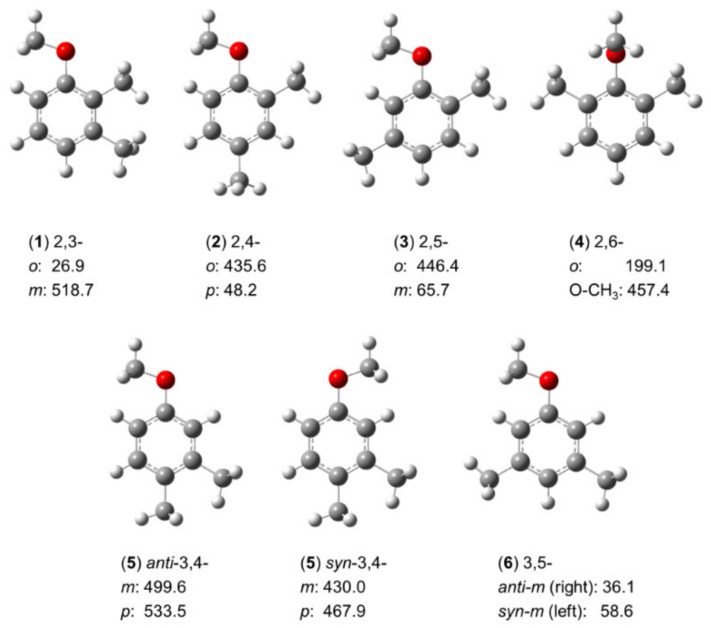
Torsional barriers of the methyl rotors in six isomers of dimethylanisole (in cm^−1^). (**1**) 2,3-dimethylanisole [84], (**2**) 2,4-dimethylanisole [82], (**3**) 2,5-dimethylanisole [198], (**4**) 2,6-dimethylanisole [198,199], (**5**) 3,4-dimethylanisole [83], and (**6**) 3,5-dimethylanisole [198].

The three isomers of mono-methyl anisole have shown that for o-methyl anisole, the syn- and anti-conformers of m-methyl anisole, and p-methyl anisole (molecules (**9**–**11**) in Figure 5), the respective barrier heights are 444.1 cm^−1^ [127], 55.8 cm^−1^ and 36.6 cm^−1^ [149,150], and 49.6 cm^−1^ [151], respectively. The conjugated double-bond system with complex electronic configuration leads to the expectation that the potential barriers of the methyl rotors in dimethyl anisoles cannot be directly derived from those of the mono-methyl anisoles. To understand these LAMs, investigations of all six conformers of dimethyl anisoles were performed, and the methyl torsional barriers are summarized in Figure 14. 

Except for the 2,6-isomer (**4**), no splittings arising from the methoxy methyl group are observed, so the molecules are two-top problems. The values of 435.6 cm^−1^ and 446.4 cm^−1^ found for the barrier height of the methyl rotor at the 2-position of 2,4- (**2**) and 2,5-dimethylanisole (**3**) are similar [82,198], reflecting well the local steric environment of these methyl groups. The 3-methyl rotor in 3,4-dimethylanisole (**5**) with its values of 499.6 cm^−1^ in the anti and 430.0 cm^−1^ in the syn configurations are intermediate; so are the values observed for the 4-methyl rotor (533.5 cm^−1^ and 467.9 cm^−1^, respectively) [83], as well as the 3-methyl rotor in 2,3-dimethylanisole (**1**) (518.7 cm^−1^) [84]. These values indicate the steric hindrance arising from the neighboring methyl group. Methyl rotors in the absence of steric hinderance such as the 4-methyl rotor in the 2,4-isomer (**2**) [82], the 3-methyl rotor in the 2,5-isomer (**3**) [198], and the 3- and 5-methyl rotors in the 3,5-isomer (**6**) are all very low [198] (less than 70 cm^−1^). An exception where the apparent presence of steric frustration fails to explain the barrier is the case of the 2-methyl group in 2,3-dimethylanisole (**1**) [84]: The methyl rotor squashed between a methoxy group and a methyl group features the lowest barrier observed in the entire family (26.9 cm^−1^), which is assumed to be caused by electronic effects with an explanation similar to that used for 2-methylthiazole in Section 2.1.1.1. Supposing that the 3-methyl group and the methoxy group are similar, the 2-methyl rotor, featuring a C_3v_ symmetry, would experience a potential based on a C_2v_ frame symmetry, similar to that of nitromethane CH_3_NO_2_ (V_6_ = 4.9 cm^−1^) [200] or toluene (V_6_ = 4.8 cm^−1^) [137,138,139,140,141,142], in which a low-hindered V_6_ potential exists. However, because the 3-methyl group and the methoxy group are not similar, the frame symmetry is out-of-balance, which might induce the small value of 27 cm^−1^ found for the V_3_ potential of the o-methyl group. The 2,6-isomer (**4**) exceptionally represents a three-top case [198,199]. Since both ortho positions are substituted by a methyl group, the methoxy group is highly sterically hindered, thereby forced to tilt out of the plane spanned by the heavy atoms of the phenyl ring by an angle of 90°. Steric hindrance often increases the barrier to internal rotation, but for the methoxy methyl group, its torsional barrier dramatically decreases to about 460 cm^−^^1^, leading to observable splittings in the microwave spectrum. The reason might be the symmetric frame to which the methyl group is attached, with the explanation similar to that supposed for 2,3-dimethylanisole (**1**).

##### 2.2.2.2. Six Isomers in the Dimethylfluorobenzene Family

**Figure 15:**
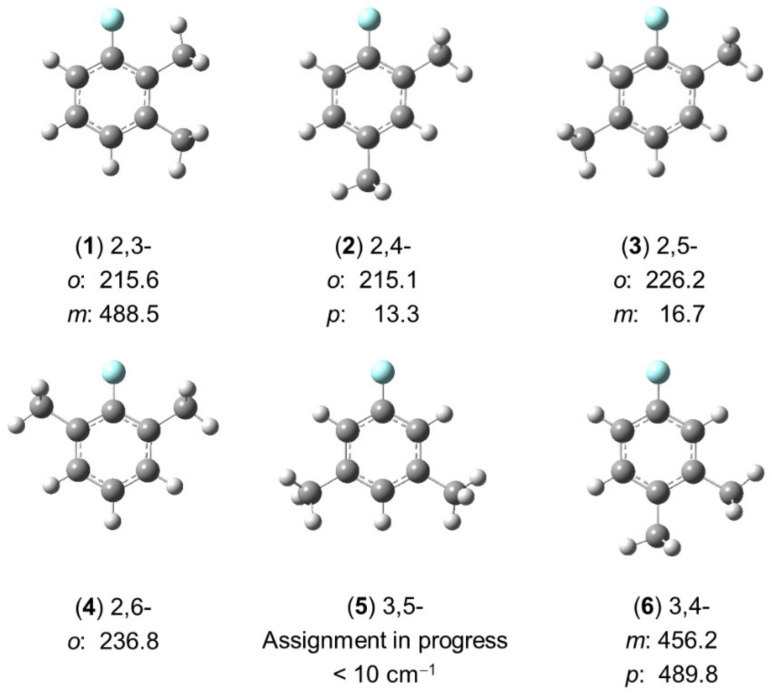
Torsional barriers of the methyl groups in six isomers of dimethylfluorobenzene (in cm^−1^). (**1**) 2,3-dimethylfluorbenzene [78], (**2**) 2,4-dimethylfluorobenzene [201], (**3**) 2,5-dimethyl-fluorobenzene [202], (**4**) 2,6-dimethylfluorobenzene [80], (**5**) 3,5-dimethylfluorobenzene, and (**6**) 3,4-dimethylfluorobenzene [79].

In the studies shown in Section 2.2.2.1 on the isomers of dimethylanisole, only for 3,4- and 2,6-dimethylanisole, in which the barriers to internal rotation of the methyl groups are intermediate, no fitting problems were encountered [83,198,199]. In the other cases, there is an argument that the vibration of the methoxy methyl group out of the C_s_ symmetry plane formed by the phenyl group cannot be neglected, and thus the Hamiltonian model has to account for it in order to accurately reproduce the rotational spectra. Since no splittings which might potentially arise from this effect were observed, the methoxy methyl group was substituted by a fluorine atom, and six isomers of dimethylfluorobenzene (shown in Figure 15) were studied to conclude whether the vibration of the methoxy methyl group is negligible or not.

Although investigations on the 3,5-isomer (**5**) are still in progress, it is already clear at this stage that the fitting problems encountered for some dimethylanisoles do not disappear for the respective isomers of dimethylfluorobenzene. The ring methyl groups behave similarly as in the cases of dimethylanisoles, except that in two isomers, 2,6- (**4**) and 3,5-dimethylfluorobenzene (**5**), the fluorine atom increases the molecular symmetry to C_2v_, while the respective isomers of dimethylanisole are C_s_. A similar situation to that observed for 2,3-dimethylanisole [84] was observed for the 2-methyl rotor of 2,3-dimethylfluorobenzene [78] with a torsional barrier being much lower than expected. The spectral assignment and fits of 3,5-dimethylfluorbenzene (**5**) are especially challenging because (i) the barrier to internal rotation of the two equivalent methyl groups is extremely low (<10 cm^−1^) and (ii) no combination difference loops could be created with only *b*-type transitions to check the assignment.

As can be recognized in Figure 15, there is almost no change in the V_3_ potential of the methyl group at the ortho position in 2,3-, 2,4-, 2,5-, and 2,6-dimethylfluorbenzene (**1**–**4**), which is adjacent to the fluorine atom. The barrier height is always about 220 cm^−1^, similar to the values found for molecules (**1**), (**4**–**6**), and (**10**–**12**) in Figure 7 (see Section 2.1.2.4). If the methyl group is only in the neighborhood of one other methyl group, then an intermediate barrier height between 450 and 500 cm^−1^ is found, similar to the values found for 3,4-dimethylanisole [83]. The steric effect is clearly the main reason for these observations. Because a methyl group is bulkier than a fluorine atom, the barrier height found for a methyl group located next to a fluorine atom is lower than the value found for a methyl group next to another methyl group. If no substituents are in the neighborhood of the methyl rotor, then the barrier to internal rotation falls to the “very low barrier” class, as in the cases of the two equivalent methyl groups in 3,5-dimethylfluorbenzene (**5**) as well as the methyl group in the para position of 2,4-dimethylfluorbenzene (**2**) [201] and the methyl group in the meta position of 2,5-dimethylfluorbenzene (**3**) [202].

### 2.3. Trimethyl- and Tetramethyl-Substituted Planar Five-Membered Rings

**Figure 16:**
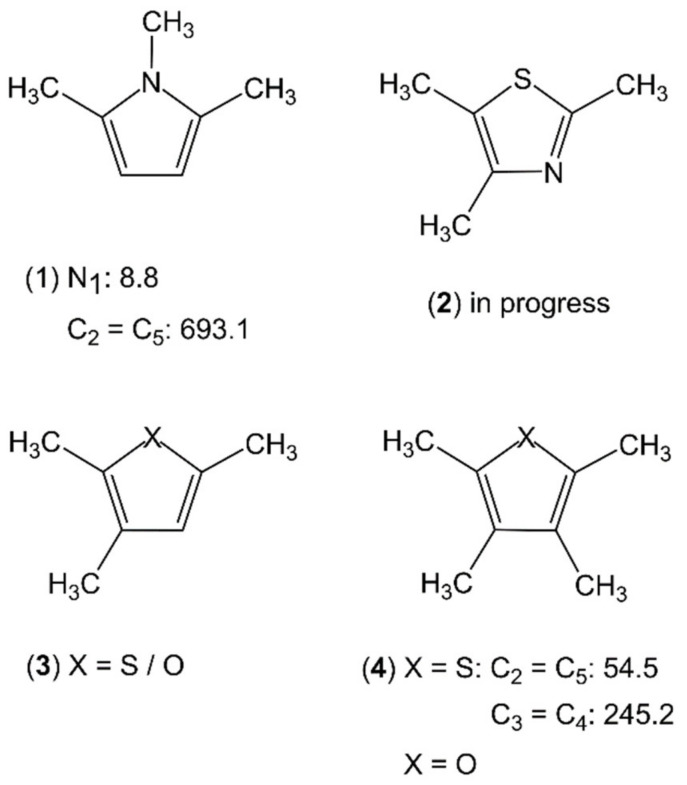
Torsional barriers of the ring methyl rotors in trimethyl- and tetramethyl-substituted planar five-membered rings (in cm^−1^). (**1**) 1,2,5-trimethylpyrrole [27], (**2**) 2,4,5-trimethylthiazole, (**3**) X = O: 2,3,5-trimethylfuran, X = S: 2,3,5-trimethylthiophene, and (**4**) X = O: 2,3,4,5-tetramethylfuran, X = S: 2,3,4,5-tetramethylthiophene [3,27].

**Figure 17:**
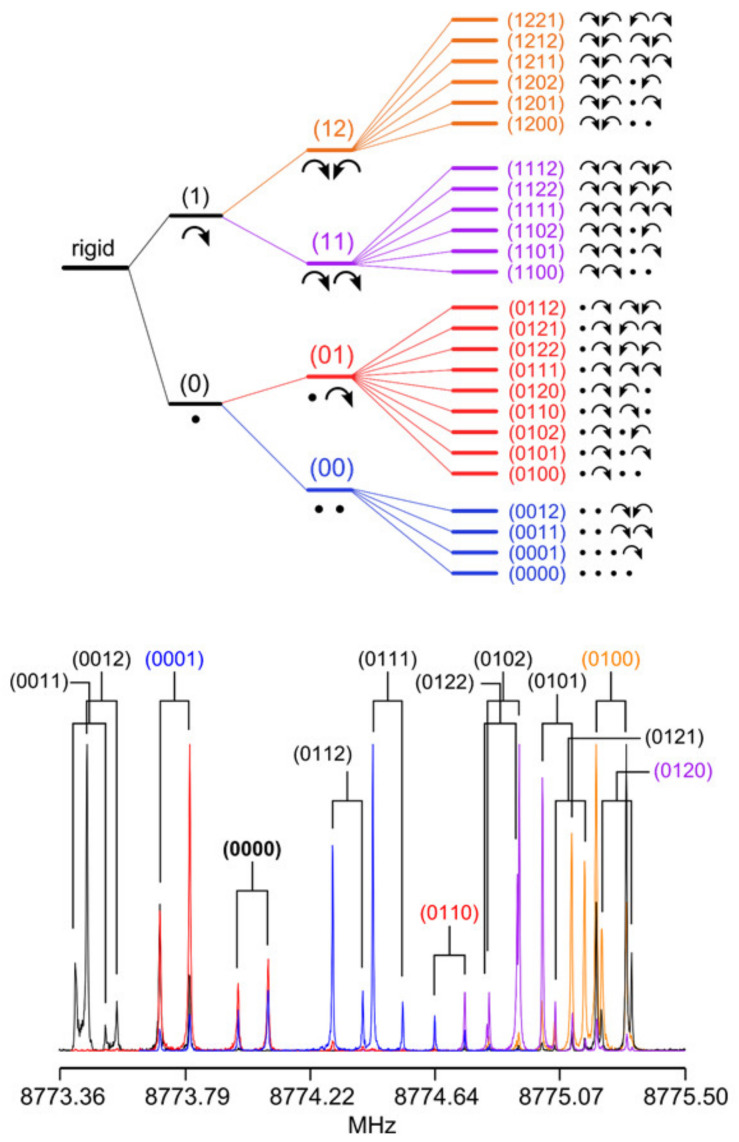
Upper figure: Torsional splittings and spin statistical weights of a rotational transition (*b*-type) of 2,3,4,5-tetramethylthiophene. Lower figure: Splittings due to internal rotation of two pairs of equivalent rotors observed in the microwave spectrum of 2,3,4,5-tetramethylthiophene [27].

Even fewer molecules belonging to the classes of trimethyl- and tetramethyl-substituted planar five-membered rings have been studied. For each class, only one example is available, which is 1,2,5-trimethylpyrrole (**1**) and 2,3,4,5-tetramethylthiophene (**4**), respectively (see Figure 16). The C_2v_ symmetry of 1,2,5-trimethylpyrrole (**1**) allows for a V_6_ potential of 8.8 cm^−1^ for the methyl group attached to the nitrogen atom and two equivalent V_3_ potentials of 693 cm^−1^ for the ring methyl groups [27]. Trimethyl-substituted thiazole (**2**), furan, and thiophene (**3**) are currently under investigation. The microwave spectrum of 2,3,4,5-tetramethylthiophene (**4**) [3,27] requires a new program code, *ntop* [82], being able to treat four-top molecules with its complex splitting patterns of two pairs of equivalent rotors, as shown exemplarily in Figure 17, as well as Separate Fits of Large Amplitude Motion Species (*SFLAMS*) [3,77]. Its oxygen analogue, 2,3,4,5-tetramethylfuran, will be another subjected four-top molecule.

## 3. Ring Inversion Tunneling

As mentioned before, though quite often appearing in van der Waals complexes, considerably fewer studies on inversion tunneling have been reported for monomers in comparison to the large number of investigations on internal rotation. Furthermore, inversion tunneling often takes place in combination with internal rotation. Only in very few molecules is the inversion tunneling not accompanied by internal rotation. Most of the studies concern the tunneling of a phenyl ring.

**Figure 18:**
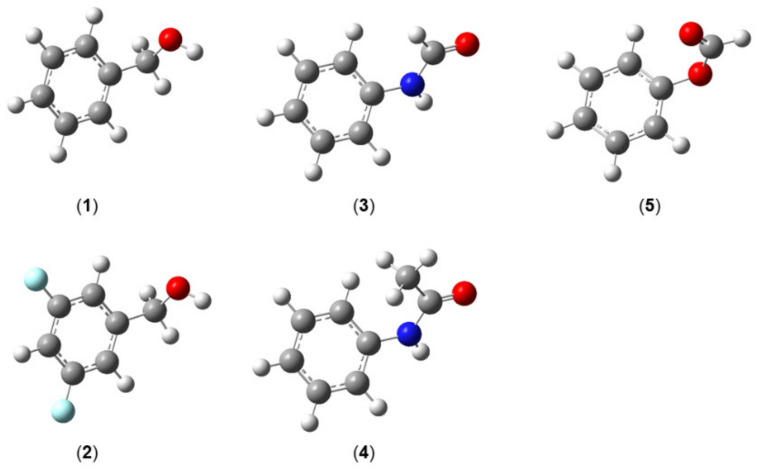
Molecules featuring an inversion-tunneling motion of the phenyl ring. (**1**) Benzyl alcohol [206], (**2**) 3,5-difluorobenzyl alcohol [207], (**3**) *E*-phenylformamide [208], (**4**) *cis*-acetanilide [209], and (**5**) phenylformate [210].

The most stable heavy-atom structure of molecules exhibiting a phenyl ring is often planar, as has been reported for molecules such as anisole [203], phenetole [204], or benzaldehyde [205]. However, in several molecules, conformers featuring C_1_ symmetry are also observed as most stable, for example in benzyl alcohol (**1**) [206] and its derivative 3,5-difluorobenzyl alcohol (**2**) [207], in which the CH_2_OH group is tilted out of the phenyl plane (see Figure 18). In those cases, there is a high possibility to observe a tunneling motion of the phenyl group. Aviles Moreno et al. reported Coriolis splittings of about 100 kHz in the microwave spectrum of *E*-phenylformamide (**3**) [208]. These splittings were interpreted by the tunneling motion of the phenyl ring between two equivalent non-planar conformations with the phenyl ring tilted out of the (NH)(CO) plane by about 40° through a transition state where the acetamide plane and the phenyl ring are perpendicular. From these splittings, the energy difference separating the inversion states was determined to be about ∆*E* = 3.732 GHz. Cabezas et al. reported similar splittings in the order of a few tens of kHz in the spectrum of *cis*-acetanilide C_6_H_5_(NH)(CO)CH_3_ (**4**) [209], where the phenyl ring tilted out of the (NH)(CO) plane by the same angle.

Phenylformate (**5**) also features a pure inversion-tunneling motion of the phenyl ring [210]. At the beginning of the investigation, phenyl formate was expected to behave as a common rigid-rotor molecule, but a rigid-rotor model has completely failed to reproduce its microwave spectra with a root-mean-square deviation of 3 MHz while the measurement accuracy was 2 kHz. Quantum chemical calculations have hinted that a state other than the ground state is populated in the molecular jet resulting from ring tunneling. This low-lying v_t_ = 1 tunneling state is calculated to lie ∆*E* = 48.24 GHz above the v_t_ = 0 ground state, corresponding to cross-state tunneling splittings on the order of about 100 GHz for all *c*-type transitions. The intra-state splittings observed for *b*-type transitions due to Coriolis interaction are up to a hundred MHz. The experimentally determined value of ∆*E* = 46.2231(25) GHz, being remarkably larger than those of *E*-phenylformamide and *cis*-acetanilide, was explained by the larger phenyl ring tilt angle of 72° in phenyl formate vs. 40° in the other two molecules.

## 4. Internal Rotation Coupled with Ring Inversion Tunneling

**Figure 19:**
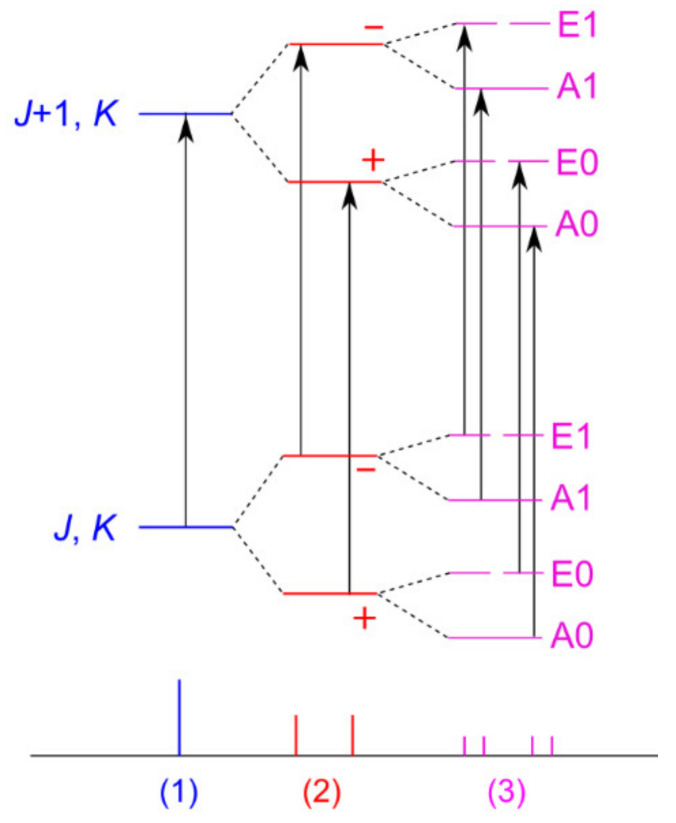
Non-scaled energy levels and microwave signal splittings for molecules featuring one two-fold tunneling motion and a methyl internal rotation, case (3), as in phenyl acetate with rotational *a*-type transitions (black arrows). Case (2) illustrates the situation of a two-fold tunneling motion without the presence of internal rotation.

This section deals with two “problematic” molecules, phenyl acetate and phenyl thioacetate, in which the internal rotation of the acetyl methyl group interacts with the tunneling motion of the phenyl ring, which is tilted out to either side of the acetyl plane, thus exhibiting a double minimum potential. All A and E torsional symmetry states are doubled due to two inversion-tunneling sub-states of different parity, and are denoted A0, A1 and E0, E1, respectively, as shown in Figure 19. Finally, we will also mention 5-methyltropolone in which the proton tunneling subsequently triggers a 60° oscillation of the methyl group and therefore couples with the methyl torsion.

**Figure 20:**
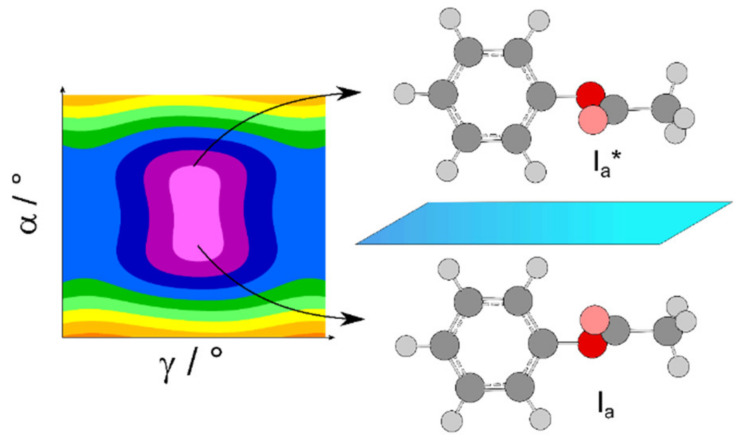
The coupled large amplitude motions of phenyl acetate. Left hand side: 1/6 of the potential energy surface calculated at the MP2/6-311++G(d,p) level of theory demonstrating the methyl and the phenyl torsions (*α* and *γ*, respectively). Right hand side: Two equivalent minima of phenyl acetate, reflecting the equilibrium configuration I_a_ and its mirror image I_a_*. The inversion-tunneling motion connects these two minima.

For phenyl acetate, the rotational spectrum was finally assigned after a long journey of more than a decade (see Scheme 1 of Ref. [3]), including all four states A0, A1, E0, and E1 [198,211,212]. The coupled LAMs are shown in Figure 20, where *α* and *γ* denote the methyl and the phenyl torsions, respectively. A global fit of the line frequencies of the four sublevels leads to the determination of the tunneling parameters ∆*E*_A0/A1_ and ∆*E*_E0/E1_ being 36.4 and 33.5 GHz, respectively. The barrier to methyl internal rotation deduced from a fit containing only A0 and E0 states is 136.4 cm^−1^, essentially the same as the value of 135.3 cm^−1^ found for isopropenyl acetate [213].

**Figure 21:**
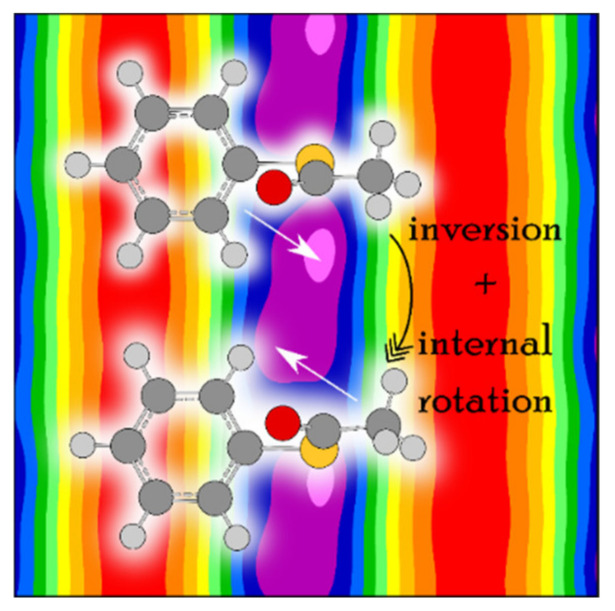
Front layer: View onto the phenyl ring of phenyl thioacetate, showing that: (i) The phenyl ring is almost perpendicular to the C–(C=O)–S plane, and (ii) there are two equivalent structures, corresponding to the inversion tunneling of the phenyl ring (or the thioacetyl group). Back layer: The potential energy surface of phenyl thioacetate calculated at the MP2/6-311++G(d,p) level of theory. The minima are extremely flat and asymmetric [198].

The coupling is stronger in phenyl thioacetate than in phenyl acetate because the bonds with the sulfur atom are less rigid than those with the oxygen atom [198,214]. Concerning the methyl internal rotation, it is generally known that methyl torsional barriers in sulfur-containing molecules are lower than those found in the oxygen analogues, as mentioned above. While the barrier of phenyl acetate is about 136 cm^−1^, the preliminary value of phenyl thioacetate is only 48 cm^−1^. This very low barrier significantly complicates the spectral assignment. Figure 21 illustrates the challenges in phenyl thioacetate with a potential energy surface in the back layer describing the coupling between two LAMs. 

**Figure 22:**
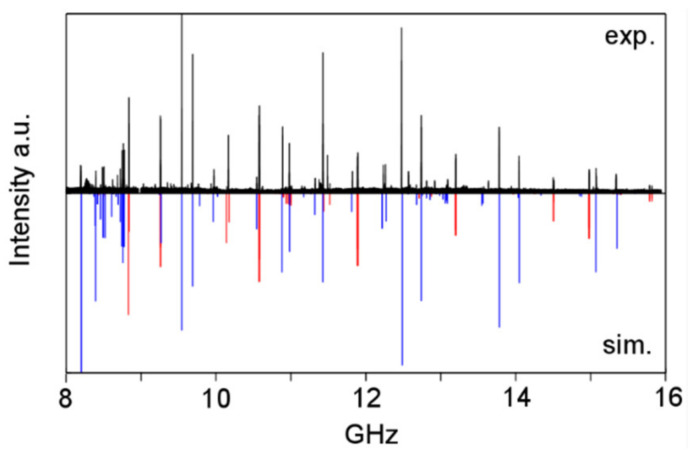
Upper trace: The broadband scan from 8 to 16 GHz of phenyl thioacetate. Lower trace: A predicted spectrum calculated using the parameters of an A0/E0 fit with a standard deviation of 3 MHz. The A species lines are in red, the E species lines in blue [198].

The spectrum depicted in Figure 22 shows that the first assignment attempts for the A0 and E0 states are most probably correct. However, extensive measurements and spectral analysis, as having been done for phenyl acetate, are required to understand the microwave spectrum of the sulfur analogue.

**Figure 23:**
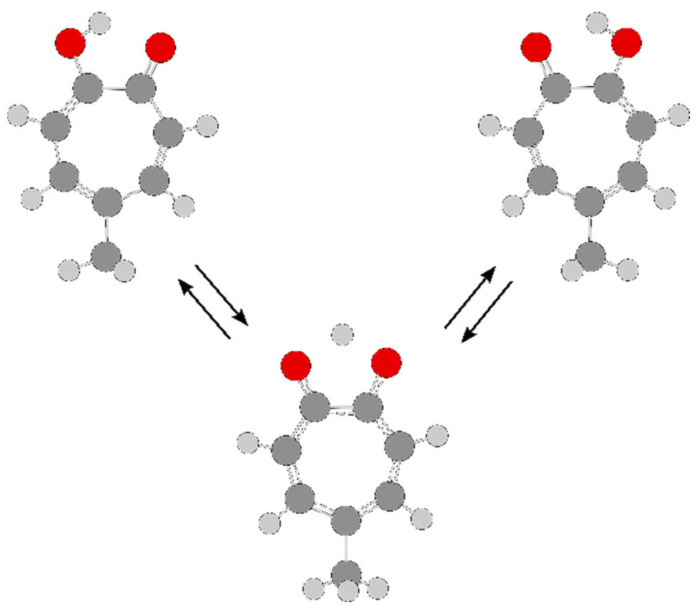
The coupled large amplitude motions of 5-methyltropolone. The two equivalent equilibrium conformations shown in the upper trace can be converted into each other over a transition state shown in the lower trace through a hydrogen-transfer tunneling path. This induces a “corrective internal rotation” [215] of 60° for the methyl torsion.

The aromatic seven-membered ring 5-methyltropolone featuring a hydroxyl and a carbonyl group at positions 1 and 2, forming a secondary five-membered cyclic structure through an internal hydrogen bond, as well as a methyl group at the position 4 of the ring (see Figure 23) is a very interesting case exhibiting LAMs coupled over a long distance [215]. The methyl group undergoes internal rotation at a barrier height of 329 cm^−1^, i.e., an intermediate value typically found for a methyl group attached to five-membered aromatic rings. The cyclic structure at the top of the molecule allows for an intramolecular hydrogen transfer due to ketone–enol tautomerism where the hydrogen atom can transfer from the hydroxyl to the carbonyl group. During this process, π-electron conjugations in the entire aromatic system rearrange, i.e., the single- and double-bond characters of CC and CO bonds are exchanged, subsequently triggering the methyl group to rotate by 60°, similar to the case of 2-methylmalonaldehyde [216]. The tautomerism strongly couples with the methyl torsion, since at equilibrium one methyl C–H bond prefers to lie adjacent to the CC double bond and on the ring plane, as illustrated in Figure 23. The splitting caused by the coupled internal motions is smaller compared to that observed for phenyl acetate and phenyl thioacetate. The quartets are often within 1 MHz, while for the two acetates A/E separations on the order of a few hundred MHz are observed. The higher methyl torsional barrier of 5-methyltropolone is the main reason for the smaller internal rotation splittings.

Ilyushin et al. studied the deuterated OD isotopologue of 5-methyltropolone, aiming at understanding whether the methyl internal rotation is dependent on subjecting mass changes in the tunneling dimension [217]. Upon OD deuteration, no counterintuitive behavior of the methyl torsion was observed. This strongly suggests that the hydrogen transfer occurs in a different time scale as that of the methyl torsion. Otherwise, the methyl internal rotation must “sense” the tunneling mass change and consequently “react” by a different torsional barrier. The same conclusion can be made by comparing the almost unchanged barrier to methyl internal rotation in phenyl acetate [212] and isopropenyl acetate [213], in which the phenyl ring of the former molecule undergoes a tunneling motion, while the isopropenyl group of the latter molecule does not.

## 5. Ring Puckering

**Figure 24:**
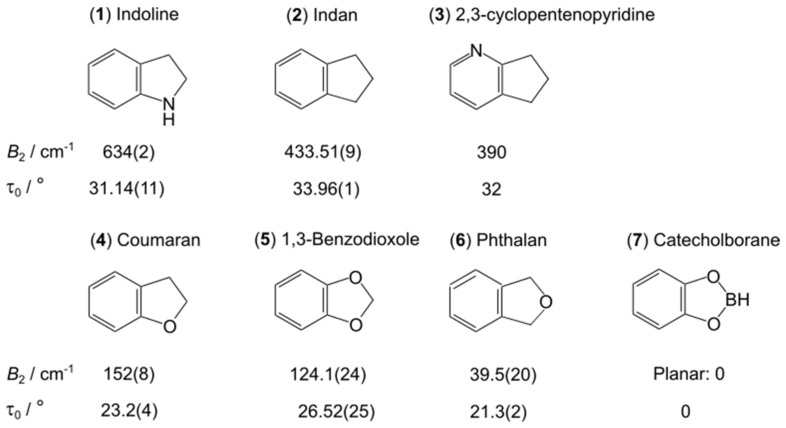
Molecules fused by an aromatic six-membered ring with an unsaturated five-membered ring with a ring-puckering motion. (**1**) Indoline [220], (**2**) indan [218], (**3**) 2,3-cyclopentenopyridine [221], (**4**) coumaran [222], (**5**) 1,3-benzodioxole [223], (**6**) phthalan [224], and for comparison the planar molecule (**7**) catecholborane [225].

Not many aromatic-ring-containing molecules featuring this kind of LAM have been studied by microwave spectroscopy. The limited amount of investigations available in the literature mainly concern an aromatic six-membered ring fused to an unsaturated five-membered ring with indan as the prototype [218] (see Figure 24). The LAM is mainly characterized by the barrier hindering the two-fold ring-puckering potential energy *B*_2_ and the equilibrium value of the puckering angle *τ*_0_, obtained in most cases using Meyer’s one-dimensional numerical flexible model [219] with the following equation:

*V*(*τ*) = *B*_2_ [1 − (*τ*/*τ*_0_)^2^]^2^. (1)

Indoline (**1**) possesses the highest *B*_2_ value of 634 cm^−1^ at *τ* = 0° (corresponding to a planar configuration) and a *τ*_0_ value of about 31° [220]. The relatively small ∆*E* value of 14.80(4) MHz was observed for the ground-state rotational transitions. Without the presence of the nitrogen atom in the five-membered ring, the barrier decreases to 433.5 cm^−1^ (∆*E* value of 22.3(2) MHz) in indan (**2**) [218] and 390 cm^−1^ in 2,3-cyclopentenopyridine (**3**) [221]. Not only the barriers hindering the ring-puckering motion but also the puckering angles are similar between molecules (**2**) and (**3**) in which the unsaturated cyclopentane ring is involved in both cases. The barrier becomes even lower with the presence of an oxygen atom in the puckering part of the molecule, with a value of 152 cm^−1^ found for coumaran (**4**, one oxygen atom) [222] and 124.1 cm^−1^ for 1,3-benzodioxole (**5**, two oxygen atoms) [223]. The ∆*E* value increases significantly to 93.682019(22) GHz and 259.726035(12) GHz, respectively. The lowest barrier is that of 39.5(20) cm^−1^ observed for phthalan (**6**) [224], in which the oxygen atom is the puckering part of the molecule instead of a CH_2_ group. Changing the CH_2_ group of 1,3-benzodioxole (**5**) to a BH group yields the planar molecule catecholborane in which the ring-puckering motion is deactivated due to the lack of two equivalent configurations [225]. Note that in all molecules presented in Figure 24, the ring-puckering LAM is coupled with a butterfly motion, and a two-dimensional model is more appropriate to obtain the *B*_2_ value than the one-dimensional model used for the comparison, but only in indoline (**1**), 2,3-cyclopentenopyridine (**3**), and 1,3-benzodioxole (**5**), this coupling has been taken into account. The *B*_2_ value given for 2,3-cyclopentenopyridine (**3**) was obtained by fixing *τ*_0_ to the ab initio value since data on the butterfly motion are not available [221]. Therefore, no errors are given.

## 6. Conclusions

Studying LAMs of molecules containing aromatic rings using microwave spectroscopy is research with great potential. For all three kinds of LAM summarized in the present review, the most characteristic parameter is the barrier hindering the LAM, which is in turn represented by splittings observed in the experimental spectrum. In general, the lower the barrier, the larger the splittings. The origin of the barrier is difficult to explain and the values are hard to predict, since chemical intuition often fails and quantum chemical calculations are not yet sufficiently accurate. For methyl internal rotation, steric and electrostatic effects both play a major role, especially because π-electron conjugations can transfer information through a longer range within the molecule. For inversion tunneling and ring puckering, the inversion or puckering angle as well as the mass of the molecular part featuring the LAM provide good indication on the order of magnitude of the splittings. Though the available investigations are exhaustive, much remains to be explored for these interesting molecular systems.

## Data Availability

Data is contained within the article.
